# Multiplexed CRISPR/Cas9-mediated knockout of 19 Fanconi anemia pathway genes in zebrafish revealed their roles in growth, sexual development and fertility

**DOI:** 10.1371/journal.pgen.1007821

**Published:** 2018-12-12

**Authors:** Ramanagouda Ramanagoudr-Bhojappa, Blake Carrington, Mukundhan Ramaswami, Kevin Bishop, Gabrielle M. Robbins, MaryPat Jones, Ursula Harper, Stephen C. Frederickson, Danielle C. Kimble, Raman Sood, Settara C. Chandrasekharappa

**Affiliations:** 1 Cancer Genomics Unit, Cancer Genetics and Comparative Genomics Branch, National Human Genome Research Institute, National Institutes of Health, Bethesda, Maryland, United States of America; 2 Zebrafish Core, Translational and Functional Genomics Branch, National Human Genome Research Institute, National Institutes of Health, Bethesda, Maryland, United States of America; 3 Genomics Core, Cancer Genetics and Comparative Genomics Branch, National Human Genome Research Institute, National Institutes of Health, Bethesda, Maryland, United States of America; Stanford University School of Medicine, UNITED STATES

## Abstract

Fanconi Anemia (FA) is a genomic instability syndrome resulting in aplastic anemia, developmental abnormalities, and predisposition to hematological and other solid organ malignancies. Mutations in genes that encode proteins of the FA pathway fail to orchestrate the repair of DNA damage caused by DNA interstrand crosslinks. Zebrafish harbor homologs for nearly all known FA genes. We used multiplexed CRISPR/Cas9-mediated mutagenesis to generate loss-of-function mutants for 17 FA genes: *fanca*, *fancb*, *fancc*, *fancd1/brca2*, *fancd2*, *fance*, *fancf*, *fancg*, *fanci*, *fancj/brip1*, *fancl*, *fancm*, *fancn/palb2*, *fanco/rad51c*, *fancp/slx4*, *fancq*/*ercc4*, *fanct*/*ube2t*, and two genes encoding FA-associated proteins: *faap100* and *faap24*. We selected two indel mutations predicted to cause premature truncations for all but two of the genes, and a total of 36 mutant lines were generated for 19 genes. Generating two independent mutant lines for each gene was important to validate their phenotypic consequences. RT-PCR from homozygous mutant fish confirmed the presence of transcripts with indels in all genes. Interestingly, 4 of the indel mutations led to aberrant splicing, which may produce a different protein than predicted from the genomic sequence. Analysis of RNA is thus critical in proper evaluation of the consequences of the mutations introduced in zebrafish genome. We used fluorescent reporter assay, and western blots to confirm loss-of-function for several mutants. Additionally, we developed a DEB treatment assay by evaluating morphological changes in embryos and confirmed that homozygous mutants from all the FA genes that could be tested (11/17), displayed hypersensitivity and thus were indeed null alleles. Our multiplexing strategy helped us to evaluate 11 multiple gene knockout combinations without additional breeding. Homozygous zebrafish for all 19 single and 11 multi-gene knockouts were adult viable, indicating FA genes in zebrafish are generally not essential for early development. None of the mutant fish displayed gross developmental abnormalities except for *fancp*^*-/-*^ fish, which were significantly smaller in length than their wildtype clutch mates. Complete female-to-male sex reversal was observed in knockouts for 12/17 FA genes, while partial sex reversal was seen for the other five gene knockouts. All adult females were fertile, and among the adult males, all were fertile except for the *fancd1* mutants and one of the *fancj* mutants. We report here generation and characterization of zebrafish knockout mutants for 17 FA disease-causing genes, providing an integral resource for understanding the pathophysiology associated with the disrupted FA pathway.

## Author summary

Deficiencies in repair of DNA damage can cause diseases such as Fanconi anemia (FA), which is characterized by birth defects, bone marrow failure, anemia, leukemia and other cancers. A set of proteins constitute the FA pathway and together orchestrate the DNA repair process. Inactivation of one or more gene(s) encoding the proteins of the DNA repair pathway in an animal model would enable us to study the functions of these proteins in maintenance of normal cellular functions and the overall health of an individual in the absence of function. We systematically targeted the FA pathway in zebrafish using CRISPR/Cas9. We generated 36 fish lines with loss-of-function mutations in 19 FA pathway genes and showed that all survive to adulthood. We did not notice obvious morphological changes except in *fancp* gene-inactivated fish, which were smaller in length. However, all mutant fish were either exclusively or in majority male. Unlike reduced fertility among FA patients, all adult mutant fish were fertile, except for the *fancd1* and *fancj* knockout males. These mutant zebrafish will serve as a huge resource for the scientific community to study the role of FA proteins in fish development, DNA repair, and as models for FA disease.

## Introduction

Fanconi anemia (FA) is a rare, mostly recessive, DNA repair deficiency disorder characterized by progressive bone marrow failure (BMF), predisposition to cancer and developmental anomalies including hypogonadism and infertility [1, 2]. About 2/3 of patients display congenital abnormalities affecting multiple organ systems including the skin, kidney and urinary tract, ears, eyes, gastrointestinal, heart, and central nervous systems. Short stature, microcephaly, microphthalmia, hypogenitalia, supernumerary or hypoplastic/absent thumb with or without absence of radius, are often observed in FA patients [3]. BMF is an inevitable consequence of FA resulting in aplastic anemia due to the depletion of hematopoietic stem cells. The age of onset of anemia is variable but typically is in the first decade [4]. Patients also develop acute myeloid leukemia (AML) or myelodysplastic syndrome that ultimately progresses to AML [4]. An increased predisposition to solid tumors, particularly head and neck squamous cell carcinoma (HNSCC), esophageal and gynecological tissues is associated with FA [1, 5]. The incidence of HNSCC in FA patients is increased 700-fold, and the onset is much earlier (in a patient’s 30s) compared to sporadic HNSCC [6]. In about 25% of patients, the first clinical presentation is AML or solid tumors [7, 8]. Thus, FA is phenotypically a heterogeneous disease [9].

Increased chromosomal instability from impaired DNA crosslinking repair upon exposure to DNA crosslinking agents such as Diepoxybutane (DEB) or Mitomycin C (MMC), is a universal cellular phenotype of patient cells and serves as an unambiguous diagnostic test for FA [3]. In humans, mutations in 22 genes are known to cause FA: *FANCA*, *FANCB*, *FANCC*, *FANCD1/BRCA2*, *FANCD2*, *FANCE*, *FANCF*, *FANCG*, *FANCI*, *FANCJ/BRIP1*, *FANCL*, *FANCM*, *FANCN/PALB2*, *FANCO/RAD51C*, *FANCP*/*SLX4*, *FANCQ/ERCC4/XPF*, *FANCR/RAD51*, *FANCS/BRCA1*, *FANCT/UBE2T*, *FANCU/XRCC2*, *FANCV/MAD2L2/REV7*, and *FANCW/RFWD3*. Our understanding of the disease is continually evolving as three of these genes were reported within the last two years [10–12]. These genes encode proteins that participate in the FA pathway (also known as FA/BRCA pathway), which orchestrates the repair of DNA interstrand crosslinks (ICL) [5, 12]. Proper function of FA proteins has been shown to be important in maintaining hematopoietic stem cells, guarding genomic integrity, and preventing tumorigenesis [6, 13]. Additional roles for FA proteins are emerging in aging [14], telomere biology [15], and selective autophagy and inflammation [16]. Animal models to help understand the molecular basis of FA clinical presentations and help explore the role of FA proteins in critical biological functions are needed.

Biochemical and genetic studies have revealed to some extent the structural and functional components of the FA pathway [6, 12, 17–23]. In general, there are four protein complexes each performing a distinct function in accomplishing the repair of the damaged DNA: core complex (FANCA, FANCB, FANCC, FANCE, FANCF, FANCG, FANCL, and FANCM), ID2 complex (FANCD2-FANCI), nucleolytic processing (FANCP and FANCQ), and homologous recombination (FANCD1, FANCJ, FANCN, FANCO, FANCR, FANCS, FANCU, and FANCW*)*. Upon recognition of a signal of DNA damage the core complex along with FANCT and the FA-associated proteins (FAAP) ubiquitinates the ID2 complex. Subsequent nucleolytic processing leads to translesion synthesis by a DNA polymerase (FANCV), and the repair process is completed by homologous recombination. Though distinct biochemical functions of most of the core complex proteins that perform ubiquitination is yet unknown, it appears that sub-complexes of FANCA-FANCG confer stability to the complex, FANCC-FANCE-FANCF offer specificity and efficiency, and FANCB-FAAP100-FANCL along with FANCT transfer ubiquitin moieties on to the ID2 complex.

Only a fraction of FA gene homologs are present in any invertebrate model organism [24, 25], limiting the utility of these models. All are present in zebrafish with the exception of *FANCS/BRCA1* [26, 27]. Zebrafish provide an excellent opportunity to understand FA associated BMF and congenital anomalies, as hematopoiesis and embryonic development in zebrafish are well studied [28] and the ability to perform high throughput mutagenesis by CRISPR/Cas9 in zebrafish [29, 30] allows us to generate targeted mutations readily in many genes. We generated loss-of-function (frameshift) mutants for these 17 FA genes: *fanca*, *fancb*, *fancc*, *fancd1/brca2*, *fancd2*, *fance*, *fancf*, *fancg*, *fanci*, *fancj/brip1*, *fancl*, *fancm*, *fancn/palb2*, *fanco/rad51c*, *fancp/slx4*, *fancq*/*ercc4*, *fanct*/*ube2t*. We also targeted two additional genes encoding FA-associated proteins (*faap100* and *faap24)*. Here, we present our data on the generation of mutant fish, evaluation of the consequences of genomic indels at the mRNA level, and characterization of mutant fish phenotypes such as growth, viability, sex differentiation, and fertility.

## Results

### Multiplexed CRISPR/Cas9-mediated mutagenesis to generate FA pathway gene knockouts

The goal of our study was to analyze the *in vivo* functions of all known FA genes. Therefore, we applied the CRISPR/Cas9 technology to generate loss-of-function mutations in zebrafish. We targeted 17 genes known to have disease-causing mutations in FA patients, and two genes encoding for FA-associated proteins (S1 Table). To maximize the chances of generating loss-of-function mutations, we selected CRISPR target sites in the first half of the coding region (S1 Fig). Based on recent studies that demonstrated efficient multiplexed mutagenesis in zebrafish using CRISPR/Cas9 [29, 30], we employed this approach to generate knockouts. Multiplexing was primarily based on the known interactions among the FA proteins and their specific roles in the FA pathway [18–21], and therefore we also generated multi-gene knockouts to study their combined effect (Table 1). Seven groups of pooled sgRNAs, that were prescreened for activity, were injected to target these 19 genes, which included three groups each of two and three genes and a group of four genes. The injected fish (mosaic founders) were screened for germline transmitting mutations in each of the co-injected genes by genotyping of embryos from their outbred progeny. By screening a total of 75 founder fish, we identified 59 germline transmitting founders that passed multiple combinations of indel mutations in the co-injected genes. About three quarters of the germline transmitting founders showed mutations in two or more genes (46/59 = 78%) (Table 1). Our data demonstrate that multiplexing of sgRNAs is an efficient approach to directly generate multi-gene mutant fish lines when using prescreened active sgRNA’s.

**Table 1:** Multiplexing scheme and efficiency of germline transmission of mutations in co-targeted genes.

Injection Group		1	2	3	4	5	6	7	Total
Role in ICL repair		FA Core Complex—Complex Stability and Chromatin Loading	FA ID2 Complex	Nucleolytic Processing	FA Core Complex—Monoubiquitination	FA Core Complex—Substrate Binding and Positioning	^$^FA Core Complex—Chromatin and DNA Targeting, Translocation, and ATR Activation	Homologous Recombination	
**Gene 1**		*fanca*	*fancd2*	*fancp*	*fancb*	*fancc*	*fancm*	*fancd1*	
**Gene 2**		*fancg*	*fanci*	*fancq*	*fancl*	*fance*	*faap24*	*fancj*	
**Gene 3**					*fanct*	*fancf*	*faap100*	*fancn*	
**Gene 4**								*fanco*	
**Founders screened**		10	14	5	10	4	12	20	75
**Positive founders**		7	8	4	10	3	9	18	59
**Founders with Mutations in**	**1 gene**	2	2	0	4	0	1	4	13
	**2 genes**	5	6	4	1	1	3	6	26
	**3 genes**				5	2	5	4	16
	**4 genes**							4	4

^$^ only Fancm and Faap24 are associated with this function. Faap100 is now known to form a stable complex with Fancb and Fancl

We outbred the selected founder fish with germline transmitting mutations to generate F1 fish heterozygous for frameshift mutations in each targeted gene. These fish were genotyped at adulthood for each of the co-targeted genes. Our selection criteria for establishing mutant lines was to use either the F1 fish heterozygous for the desired mutant alleles in multiple genes or the F1 fish heterozygous for specific mutant alleles while carrying WT alleles at all other co-injected loci. We excluded any fish that were heterozygous for the desired allele but carried a non-desirable mutant allele, such as an in-frame indel mutation at the other co-injected loci, to avoid extensive genotyping in future generations. We selected two frameshift mutations for each gene, except one each for *fancb* and *fancd1*, resulting in a total of 36 single gene mutant alleles for the follow-up phenotypic analyses (S2 Fig, S2 Table). Through our multiplexing effort, we were able to generate nine multi-gene mutant allele combinations across all seven injection groups (S3 Table). Details of the selected mutant alleles including the size of indel, predicted cDNA and protein changes, and their designation by The Zebrafish Information Network (https://zfin.org/action/feature/line-designations) are provided in S2 Table. In the subsequent sections, we refer to the mutant alleles by their “hg” nomenclature as listed in S2 Table.

### RNA analysis confirms the frameshift mutations in all knockouts and identifies aberrant splice variants in some

**Fig 1:**
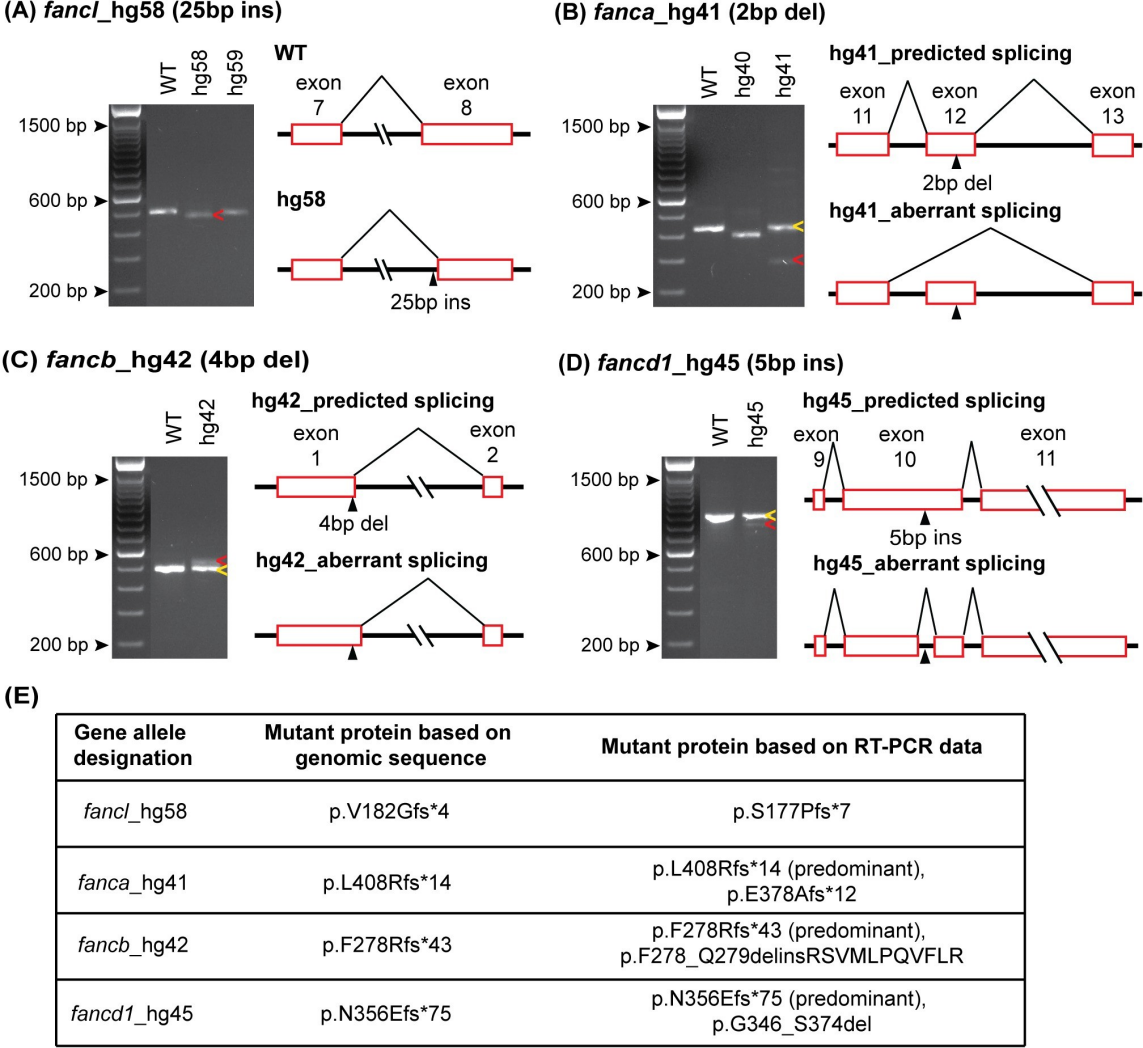
RT-PCR confirms indel mutations and identifies aberrant splice variants caused by CRISPR/Cas9 mutation. (A-D) WT and mutant allele RT-PCR products resolved on 2% agarose gel are shown in the left panels and splicing aberrations are depicted in the right panels. (A) The observed RT-PCR product for *fancl*_hg58 mutant was smaller in size (red arrow) than expected based on the 25 bp insertion in exon 8. The sequence of the hg58 product showed shift in its splice acceptor site resulting in loss of 48 bp sequence, that includes 23 bp from exon 8 beginning and CRISPR/Cas9 induced 25 bp insert. (B-D) Partial activation of cryptic splice site near the indel mutation was observed in *fanca*_hg41, *fancb*_hg42 and *fancd1*_hg45 mutant alleles. The RT-PCR products for these mutants showed two bands, one matched expected size (yellow arrows) and the other was from an altered splice product (red arrows). PCR products were cloned and sequenced to determine aberrant splice product sequence. In hg41 mutant, the indel mutation (2 bp del) in exon 12 results in partial skipping of mutated exon (B). In hg42 mutant, the indel mutation (4 bp del) in exon 1 results in partial use of new splice donor site in intron 1 (C). In hg45 mutant, the indel mutation (5 bp ins) in exon 10 results in partial use of new splice acceptor and donor in exon 10 (D). (E) The consequence on the predicted encoded protein based on the genomic indel mutation, and the observed aberrant splice product for all four mutant lines are shown. RT-PCR gel image data for all 36 alleles is shown in S3A Fig.

An indel in the genomic sequence that is not a multiple of three is predicted to cause a frameshift in translation, therefore we confirmed the presence of each predicted mutation in the mRNA transcripts. This effort would also reveal aberrant splicing caused by indel variants, if any. To this end, we performed RT-PCR for all mutant alleles using RNA extracted from adult WT and homozygous mutant fish. Our RNA analysis showed the following: 1) All 36 mutant alleles yielded an RT-PCR product (S3A Fig). 2) Products of predicted size, one wild-type and one including the indel mutation, were observed in all but one mutant allele, hg58 (Figs 1A and S3A). 3) An additional product was observed in three mutant alleles: hg41, hg42, and hg45 (Fig 1B–1D). Details of splicing aberrations in these 4 cases are described below.

The RT-PCR product from the *fancl*^*hg58/hg58*^ mutant was smaller than the expected size, based on the CRISPR/Cas9-induced 25 bp insertion mutation in exon 8 (Fig 1A). Sequencing revealed that the 25 bp insertion created a cryptic splice acceptor site leading to the deletion of 23 bp in the mRNA, resulting in a different frameshift mutation than predicted, still likely to cause a loss-of-function (Figs 1E and S3B).

The RT-PCR products from *fanca*^*hg41/hg41*^, *fancb*^*hg42/hg42*^, and *fancd1*^*hg45/hg45*^ revealed a second band, in addition to the expected band, indicating partial activation of a cryptic splice site near the indel mutation (Fig 1B–1D). The intensity of the additional band in all three mutants was weaker, suggesting low abundance of the altered splice product. Nevertheless, it might be enough to have an effect on the phenotype by generating a low level of functional protein. To determine the effect of altered splicing on the reading frame and the encoded protein, we analyzed these RT-PCR products by cloning and sequencing. In the *fanca*^*hg41/hg41*^ mutant, the additional product was missing exon 12, which contained the 2bp deletion mutation, due to altered splicing (Figs 1B and S3C). The altered splice product still generated a frameshift mutant protein (p.E378Afs*12) (Fig 1E). In the *fancb*^*hg42/hg42*^ mutant, the additional product had an insertion of 31 bp from intron 1 (Figs 1C and S3D), due to aberrant splicing caused by a cryptic splice donor in intron 1. The combined effect of a 4 bp deletion mutation near the end of exon 1 and the retention of 31 bp in the region adjacent to intron 1 generated an in-frame mutant protein with a deletion of 2 amino acids and an insertion of 11 unrelated amino acids (p.F278_Q279delinsRSVMLPQVFLR). The additional product in the *fancd1*^*hg45/hg45*^ mutant had a deletion of 92 bp in exon 10 (includes 87 bp from WT sequence and a 5 bp insertion mutation) (Figs 1D and S3E). The aberrant splice product generated an in-frame mutant protein with a deletion of 29 amino acids (p.G346_S374del). The in-frame mutant proteins generated by *fancb*^*hg42/hg42*^ and *fancd1*^*hg45/hg45*^ could potentially maintain their function (Fig 1E). The unintended consequences of CRISPR/Cas9-induced indel mutations such as aberrant splicing, that we observed in our zebrafish mutants, highlight the importance of analyzing the RNA, rather than relying on genomic DNA analysis alone. This is particularly important if the mutant fish do not display any phenotype.

### The frameshift mutant alleles are indeed null alleles

It is important to validate that the frameshift mutant alleles lead to generation of truncated proteins as predicted and are therefore true loss-of-function alleles. However, due to the absence of zebrafish antibodies or cross-species reacting antibodies for nearly all FA proteins, we could not evaluate protein expression in our mutants, except for Fancd2. Finding that a human FANCD2 antibody recognized its zebrafish counterpart, allowed us to confirm that the Fancd2 is not expressed in both *fancd2* knockout lines, indicating that these are indeed null alleles (S4 Fig).

As an alternate method, we used a recently described functional fluorescent mutation reporter assay [31] to test mutant alleles for a subset of genes: *fance* (hg48), *fancf* (hg50), *fancg* (hg52), *fancl* (hg59) and *fanct* (hg70). Despite robust control RFP expression, lack of GFP expression driven by the mutant allele, when compared to the WT allele, indicated that these frameshift mutations indeed introduce a premature stop codon (S5 Fig). These data show that the mutant alleles for all five genes tested are true loss-of-function frameshift mutations. In addition, as described below, we demonstrated hypersensitivity of nearly all frameshift mutant alleles to DEB treatment indicating that these are indeed null alleles.

### Homozygous knockout embryos display hypersensitivity to DEB treatment

**Fig 2:**
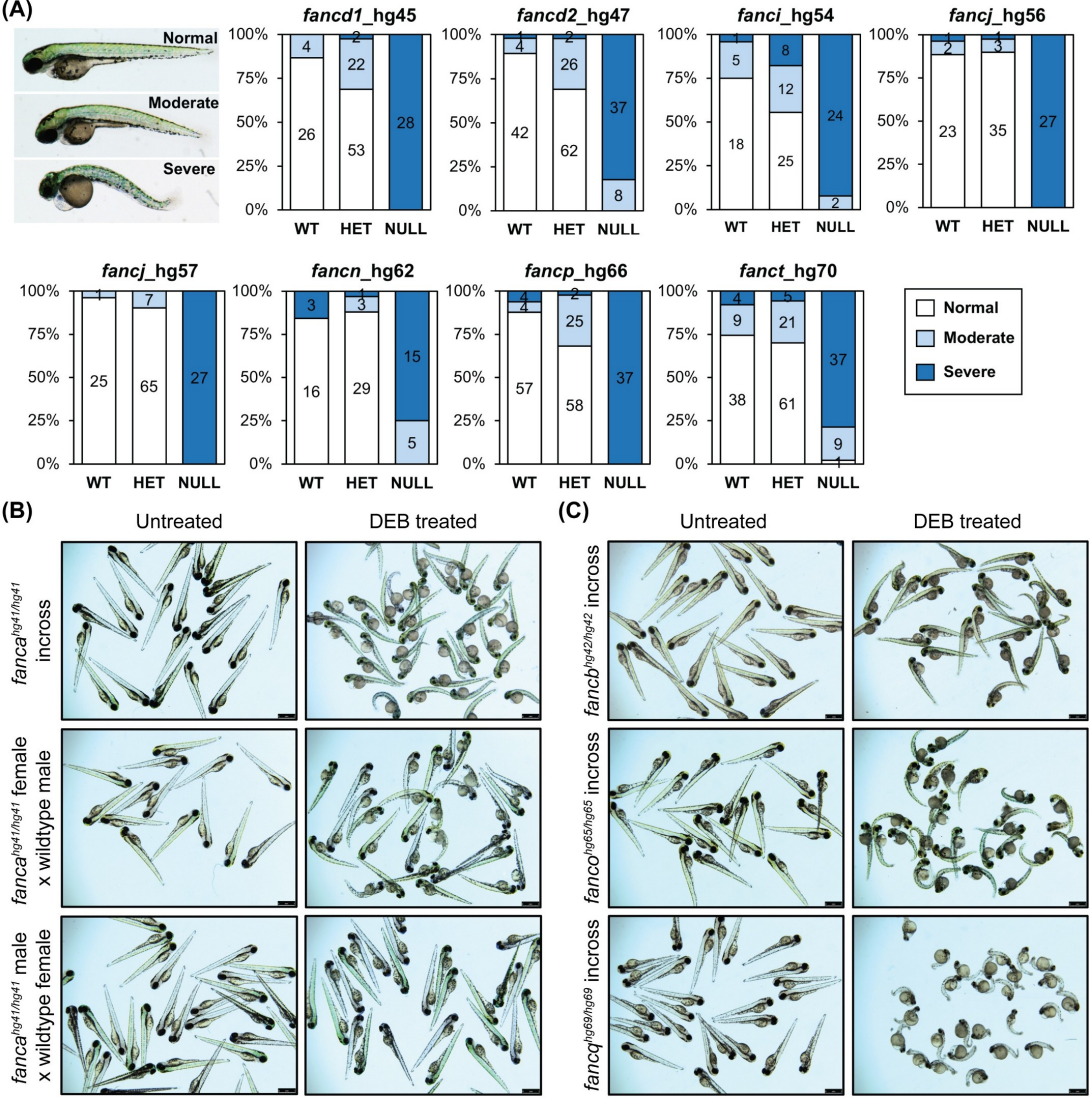
Homozygous knockout mutants are sensitive to DEB treatment. (A) Embryos obtained from inbreeding heterozygous knockouts of *fancd1* (hg45; 0.9 μg/mL DEB), *fancd2* (hg47; 0.9 μg/mL DEB), *fanci* (hg54; 0.65 μg/mL DEB), *fancj* (hg56 and hg57; 0.6 μg/mL DEB), *fancn* (hg62; 0.8 μg/mL DEB), *fancp* (hg66; 0.9 μg/mL DEB) and *fanct* (hg70; 0.8 μg/mL DEB) were treated at indicated DEB concentrations between 4–72 hpf. Treated embryos were classified based on severity of morphological changes observed into three phenotypic groups: normal (WT appearance), moderate (slight body curvature and minor edema) and severe (emaciated appearance, severe body curvature and large edema). An example image of DEB treated embryo (72 hpf) for each group is shown on the left. Distribution of each phenotypic group for a given genotype are displayed as stacked bar chart. The segments in bar show percent of embryos in each morphological group: normal (white), moderate (light blue), severe (dark blue). The number in each segment depicts the number of embryos for a given phenotypic group. (B) Maternal WT *fanca* transcript rescues embryos from DEB hypersensitivity. Embryos obtained from indicated *fanca*_hg41 breeding were treated with DEB (0.8 μg/mL). Representative images show untreated and DEB treated embryos. (C) Embryos generated from inbreeding of homozygous knockouts of *fancb* (hg42; 0.8 μg/mL DEB), *fanco* (hg65; 0.8 μg/mL DEB) and *fancq* (hg69; 0.5 μg/mL DEB) were treated at indicated DEB concentrations. Representative images show untreated and DEB treated embryos.

FA patient cells show hypersensitivity to DNA cross-linking agents such as DEB and MMC resulting in chromosomal breakage [3]. To determine if our mutants also exhibit similar hypersensitivity, the embryos from inbred heterozygous mutants were treated with DEB. The homozygous knockouts from *fancd1* (hg45), *fancd2* (hg47), *fanci* (hg54), *fancj* (hg56 and hg57), *fancn* (hg62), *fancp* (hg66) and *fanct* (hg70) mutant lines showed severe morphological changes compared to their WT and heterozygote clutch mates, indicating their hypersensitivity to DEB (Fig 2A). However, similar hypersensitivity was not distinct for the remaining mutants, possibly due to the protection provided by wildtype maternal transcript. To test this, we set up *fanca* (hg41) homozygous mutant incross, as both male and female were available, and also outcross with wildtype counterparts. Untreated embryos from all three crosses looked normal. Hypersensitivity to DEB treatment was observed among all the embryos from incross, and most embryos from female mutant outcross despite all being heterozygotes. But, none of the embryos from male mutant outcross were affected indicating that the presence of wildtype *fanca* maternal transcript rescues embryos from hypersensitivity to DEB treatment (Fig 2B). Additionally, we incrossed homozygous knockout mutant fish for *fancb* (hg42), *fanco* (hg65) and *fancq* (hg69), and observed similar hypersensitivity of the embryos to DEB treatment (Fig 2C). The lack of null females due to sex reversal phenotypes (described later) prevented us from preforming inbreeding experiments for other alleles. Overall, we demonstrate homozygous mutant embryo specific DEB sensitivity for 11 out of 17 FA gene mutants either by heterozygote or homozygote inbreeding. Nevertheless, our DEB test results further validate all the tested knockout mutants indeed have lost function of the targeted gene.

### All single gene knockouts survive to adulthood

**Fig 3:**
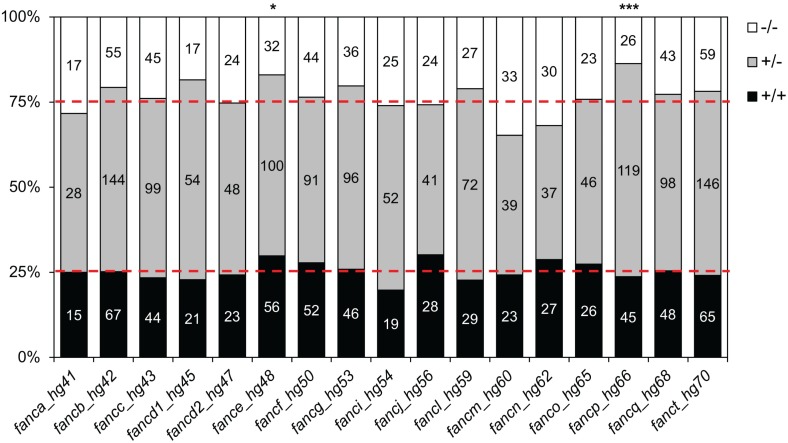
Homozygous knockout mutant fish for FA genes survive to adulthood. Progeny from inbred heterozygous fish for each allele were genotyped at 3–6 mpf. Data are shown as stacked bar chart, where each bar represents one mutant allele, as marked on the X-axis by the gene name and hg#. Segments on the bar show % of fish in each of the three expected genotypes: +/+, +/-, and -/- as marked on the Y- axis. Numbers in each segment depict the number of fish for each genotype. Data from one allele for each gene are shown here and the other allele is shown in S6 Fig. Reduced adult survival was observed in *fance*^*hg48/hg48*^ and *fancp*^*hg66/hg66*^ fish (Chi-square analysis, *p < 0.05, ***p < 0.001, respectively).

**Fig 4:**
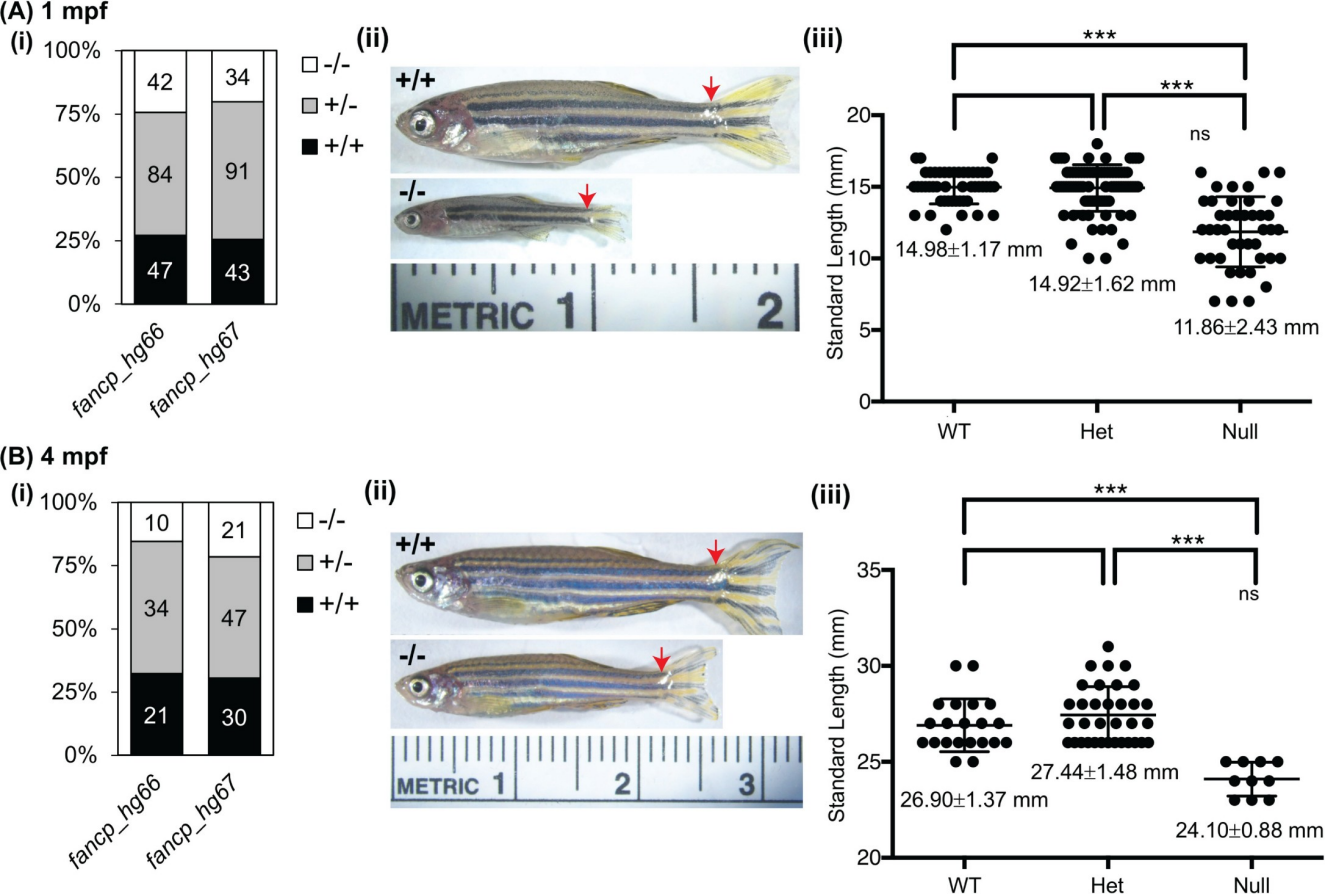
Homozygous *fancp* mutants are significantly smaller in body length than their siblings. Standard length measurements of *fancp* fish at 1 mpf (A), and 4 mpf (B). (i) Number of fish genotyped and measured for both *fancp* alleles: hg66 and hg67. (ii) Representative images of *fancp*^*+/+*^ and *fancp*^*hg66/hg66*^ fish with red arrows marking the beginning of caudal fin used in length measurements. (iii) Data on body size measurements for *fancp*^*+/+*^, *fancp*^*hg66/+*^ and *fancp*^*hg66/hg66*^ fish. Both time points show a significant decrease in size of *fancp*^*hg66/hg66*^ fish compared to the WT and heterozygous clutch mates (ANOVA analysis, ***p<0.001). Data for *fancp*_hg67 is shown in S8 Fig.

To determine if the homozygous knockouts survive to adulthood, we grew progenies from pairwise heterozygous crosses of all mutations to adulthood and determined the genotypes of the surviving fish. Homozygous knockout fish were observed among the surviving adults for all genes, indicating no lethality at earlier developmental stages for the generated alleles. Furthermore, the survival was at the expected Mendelian ratios for the majority of the targeted genes. In *fancp*, *fance*, and *faap24*, however, we observed discordant results between the survival of homozygous fish for the two different mutant alleles, where survival of one of the two knockout alleles was consistent with a Mendelian ratio while in the other allele it was not (Figs 3, S6 and S7). Reduced survival was statistically significant for *fancp*^*hg66/hg66*^ (p = 0.0003) but not for *fancp*^*hg67/hg67*^ (p = 0.0875) fish. Genotyping at one month post fertilization (mpf) revealed that the number of *fancp*^*hg66/hg66*^ homozygous fish at this age were consistent with Mendelian ratio (Fig 4A(i)), indicating that some of the *fancp*^*hg66/hg66*^ fish die between 1 mpf and adulthood. Similarly, *fance*^*hg48/hg48*^ (p = 0.011) and *faap24*^*hg75/hg75*^ (p = 0.027) fish showed reduced survival, while *fance*^*hg49/hg49*^ fish (p = 0.077) and *faap24*^*hg74/hg74*^ (p = 0.079) survived in expected numbers (S7 Fig). Overall, our data suggest knockouts of individual genes from FA pathway in zebrafish are not lethal.

### Multi-gene knockouts survive to adulthood

Multiplexing enabled us to obtain fish with mutations in multiple genes directly from founder fish outcrosses. Theoretically, twenty-six combinations of double, triple, and quadruple gene F1 heterozygous knockouts were possible from the seven injection groups (S3 Table). However, our selection criteria were to retain and analyze only those carrying the mutant alleles described for single gene knockouts (S2 Table). Nevertheless, we obtained nine combinations of multi-gene F1 heterozygous mutants. Six of these were inbred to generate multi-gene homozygous knockouts, while the remaining three were not inbred due to the absence of either male or female carriers. Our data showed that all 11 possible multi-gene homozygous knockout fish were adult viable, suggesting absence of epistasis in the knockouts for tested gene combinations (S3 Table).

### *fancp*^*-/-*^ fish have smaller body length

During the adult survival experiment, we observed that the *fancp*^*-/-*^ adult fish for both mutations were smaller in size than their WT and heterozygous clutch mates. To follow up on this observation, inbreedings for *fancp*^*hg66/+*^ and *fancp*^*hg67/+*^ were done in duplicate and their progeny’s body length were measured at juvenile (1 mpf) and adult stages (4 mpf). The homozygous knockout fish with both alleles were significantly smaller in body length than their clutch mates at 1 and 4 mpf (Figs 4A and 4B, S8A and S8B). The observed smaller body length phenotype in *fancp*^*-/-*^ appears to reflect the short stature frequently observed in FA patients.

To examine the effect of *fancq* mutant alleles on smaller body length of *fancp* mutant alleles, we inbred double heterozygotes *(fancp*^*hg66/+*^;*fancq*^*hg68/+*^) and measured body lengths of their progenies. All *fancp* homozygous knockout fish, irrespective of *fancq* genotype, were significantly smaller, indicating the *fancq* mutation has no role in expression of this phenotype (S9 Fig).

### All FA gene knockouts exhibit varying levels of female-to-male sex reversal phenotype

**Fig 5:**
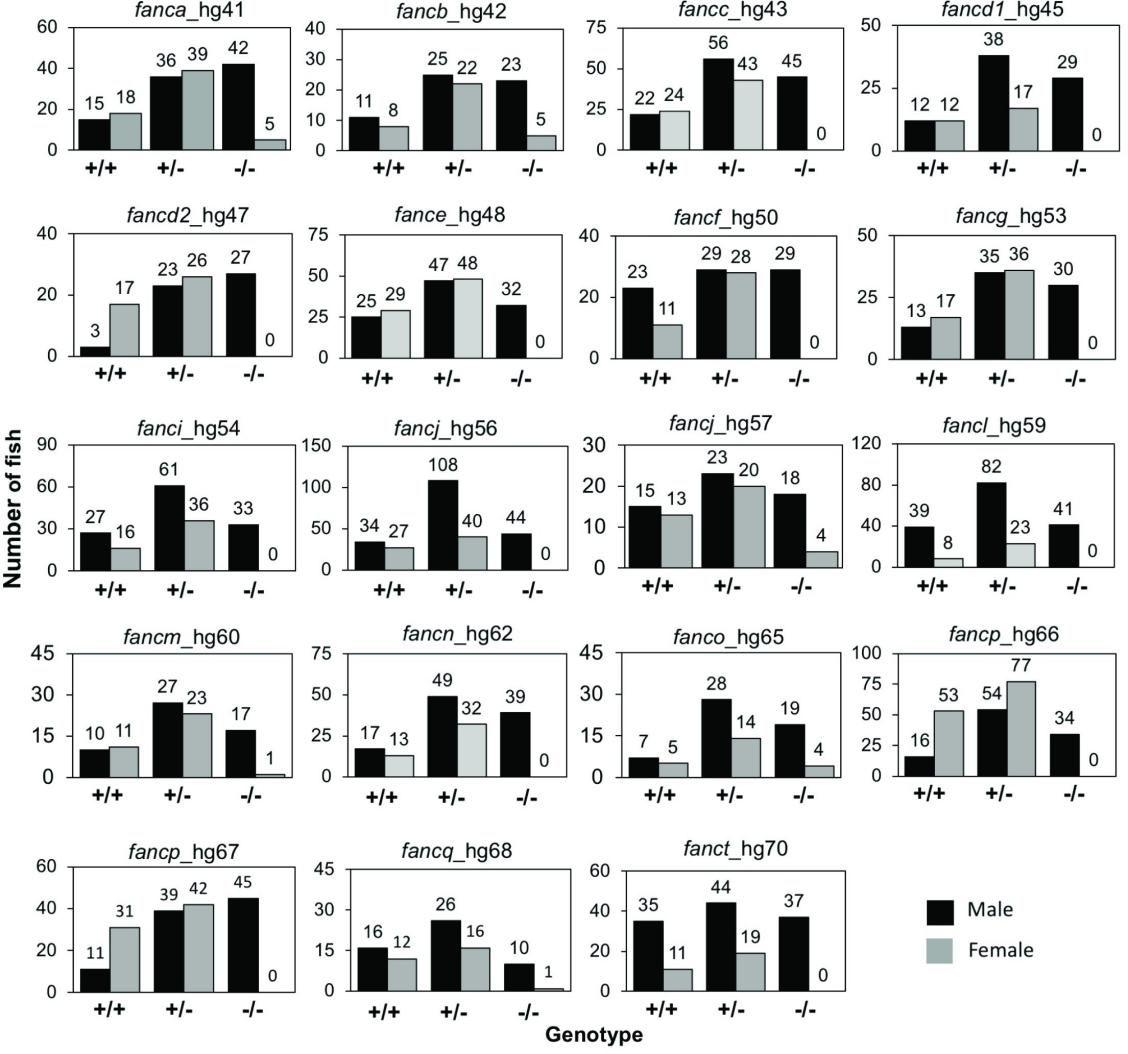
Female-to-male sex reversal was observed in FA gene knockouts. Progenies from inbred heterozygous fish for each gene were genotyped around 3 months of age and the sex was determined. For each gene, the data is shown as bar graph for number of male and female fish in each genotype category as marked on the X-axis. Numbers at the top of bar depict the number of fish. Significantly increased number of males were observed for *fanca*, *fancb*, *fancm*, *fanco*, and *fancq* homozygous knockouts, while only males were observed for the rest. For *fancj*^*-/-*^, no females were observed in *fancj*^*hg56/56*^, whereas greatly reduced number of females were observed in *fancj*^*hg57/hg57*^.

Zebrafish have high developmental plasticity for sex determination and they lack the sex-determining chromosome(s) of mammals [32]. The plasticity of sex determination in zebrafish can help us to study mechanisms and factors that are associated with gonadogenesis and hypogonadism. We raised progeny from inbred heterozygous fish from one allele for each FA gene (alleles shown in Fig 3) and sexed them after genotyping at 3–4 mpf. Surprisingly, for 12 FA genes (*fancc*, *fancd1*, *fancd2*, *fance*, *fancf*, *fancg*, *fanci*, *fancj*, *fancl*, *fancn*, *fancp*, and *fanct*), no females were observed among surviving homozygous knockouts, and for the remaining five FA genes (*fanca*, *fancb*, *fancm*, *fanco*, and *fancq*), homozygous knockout females were in greatly reduced numbers (Fig 5). The presence of only males, or significantly increased number of males, in adult knockouts (Fig 5) and the absence of reduced survival of adult knockouts (except for *fancp*^*hg66/hg66*^) (Figs 3 and S6) suggests a female-to-male sex reversal phenotype among FA gene knockout fish. We tested both alleles for *fancp* due to reduced body length, and allele specific discrepancy in their survival. The female-to-male sex reversal phenotype was observed in both *fancp* lines (Fig 5). We also tested both *fancj* alleles as they displayed a different male fertility phenotype (as described in the section below). Interestingly, the *fancj*^*hg56/hg56*^ showed a complete female-to-male sex reversal, whereas the *fancj*^*hg57/hg57*^ allele showed a partial sex reversal phenotype (Fig 5). It is possible that the partial sex reversal phenotypes observed for *fanca*, *fancb*, *fancm*, *fanco*, and *fancq* may also be allele specific. To test this, we checked other mutant allele lines for these genes except for *fancb*, for which we only had one mutant allele. Second mutant alleles for *fanca*, *fanco* and *fancq* also revealed the partial sex reversal phenotype, whereas no females were observed in the second *fancm* mutant line indicating complete sex reversal phenotype (S10 Fig). Overall, our data show that the FA genes are important for gonadogenesis in zebrafish, which may reflect the commonly observed hypogonadism among FA patients [2, 3].

To determine the stage at which FA gene mutations affects sex differentiation, we examined histological sections of larval (21 dpf) and juvenile (45 dpf) stage gonads of *fancc*_hg43 mutant fish as a representative mutant allele. At 21 dpf, the gonads of both homozygous knockout and heterozygous fish were undifferentiated and contained gonocytes. By 45 dpf, a definitive testicular differentiation was apparent among homozygotes, whereas both ovarian or testicular differentiation was apparent among heterozygotes (S11 Fig).

### Loss of *tp53* rescues ovarian development in mutant fish

**Fig 6:**
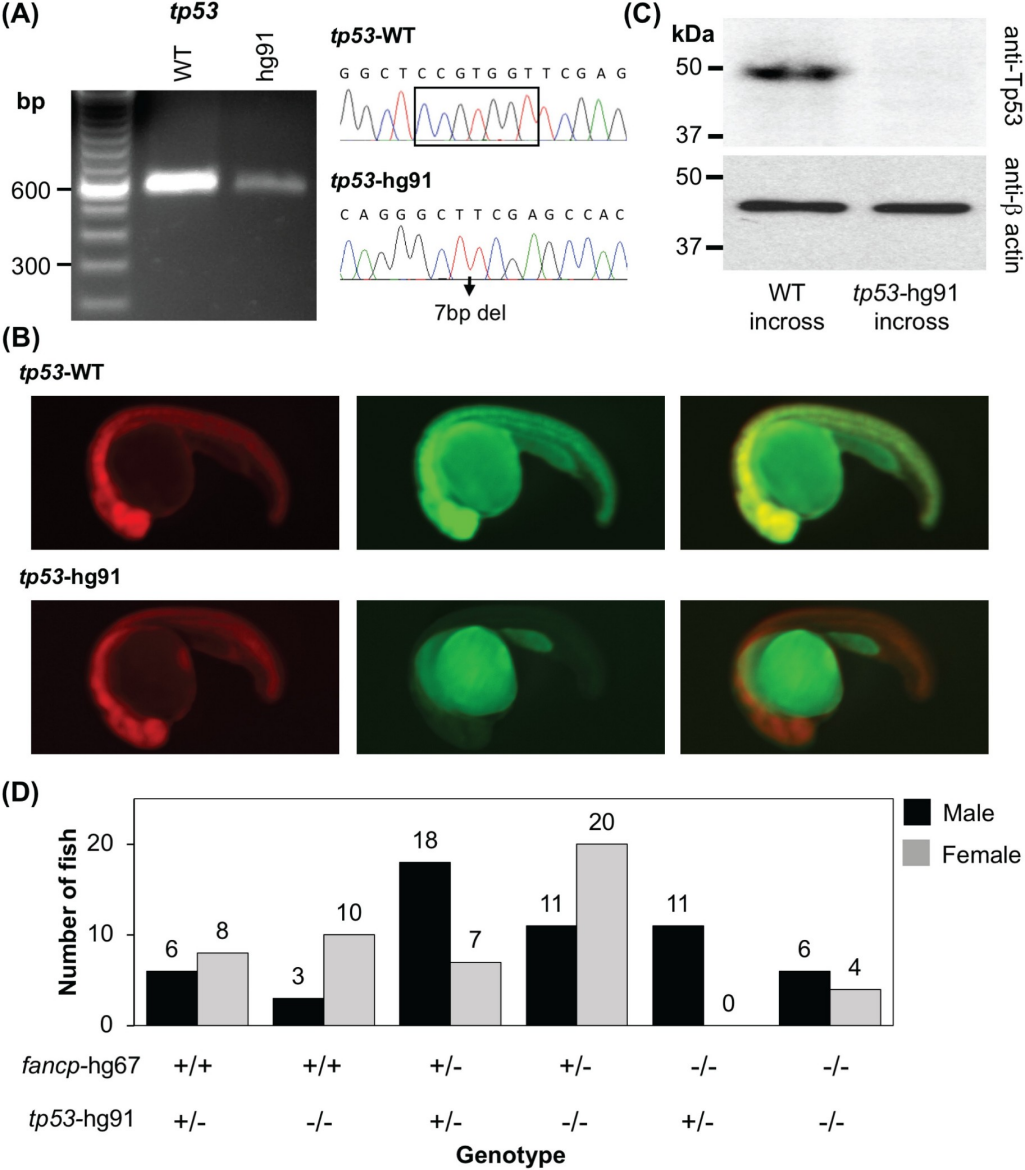
Co-mutation in *tp53* gene rescues female-to-male sex reversal of *fancp* homozygous knockouts. (A) RT-PCR confirmed the absence of any aberrant splice variants around *tp53* gene mutation. Sanger sequencing confirmed the presence of CRISPR-Cas9 introduced 7bp deletion mutation. The products were resolved on 2% agarose gel. (B) Reporter assay to check the expression of *tp53* frameshift mutant. Representative images at 1 dpf of embryos co-injected with the specified reporter mRNA and TagRFP are shown as RFP (left panel), GFP (middle panel) and merged (right panel). (C) Western blot analysis shows absence of Tp53 protein in the knockout embryo extracts. Twenty-four hpf embryos collected from *tp53*^*hg91/hg91*^ homozygous knockout fish incross were used to check the loss of expression of Tp53 protein. Embryos obtained from TAB5 incross were used as WT controls. Expression of β-actin was used as loading control. (D) Progenies from *fancp*^*hg67/+*^;*tp53*^*hg91/+*^ and *fancp*^*hg67/+*^;*tp53*^*hg91/hg91*^ breeding were genotyped around 4 mpf and the sex was determined. Number of male and female fish in each genotype category is presented. The genotypes are marked on the X-axis. Among the *fancp* homozygous knockouts, only males were observed with *fancp*^*hg67/hg67*^;*tp53*^*hg91/+*^ genotype, whereas both males and females were observed with *fancp*^*hg67/hg67*^;*tp53*^*hg91/hg91*^ genotype.

To examine if loss of *tp53* can rescue the sex reversal phenotypes as previously demonstrated for *fancd1*, *fancl* and *fancr* [33–36], we introduced *tp53* knockout mutation into *fancp* mutant fish as a representative of FA gene mutants with complete female to male sex reversal. First, *tp53* mutant (hg91: c.368_374delCCGTGGT; p.S123Ffs*38) fish were generated using CRISPR-Cas9 method to target exon 5. We deliberately targeted exon 5 to generate a frameshift indel mutant that should result in premature termination in all known *tp53* transcript isoforms [37, 38]. RT-PCR and fluorescent mutation reporter assay confirmed that the frameshift caused by 7bp deletion results in premature termination (Fig 6A and 6B). Furthermore, the availability of zebrafish Tp53 antibody allowed us to confirm that our *tp53* mutant allele is indeed a null mutant (Fig 6C). To test the effect of the *tp53* null mutation on *fancp* sex reversal phenotype, we crossed *fancp*^*hg67/+*^;*tp53*^*hg91/h91*^ mutant fish with *fancp*^*hg67/+*^;*tp53*^*hg91/+*^ fish. The resulting progenies were grown to adulthood to determine the correlation between the sex and genotype of the fish. Both male and female fish were observed with *fancp*^*hg67/hg67*^;*tp53*^*hg91/hg91*^ genotype (Fig 6D), indicating that Tp53-mediated apoptosis of germ cells causes the sex reversal in *fancp* homozygous knockouts and may be a common mechanism of sex reversal phenotype in FA knockout fish.

### Homozygous knockout males for *fancd1* and *fancj* display fertility defects

**Fig 7:**
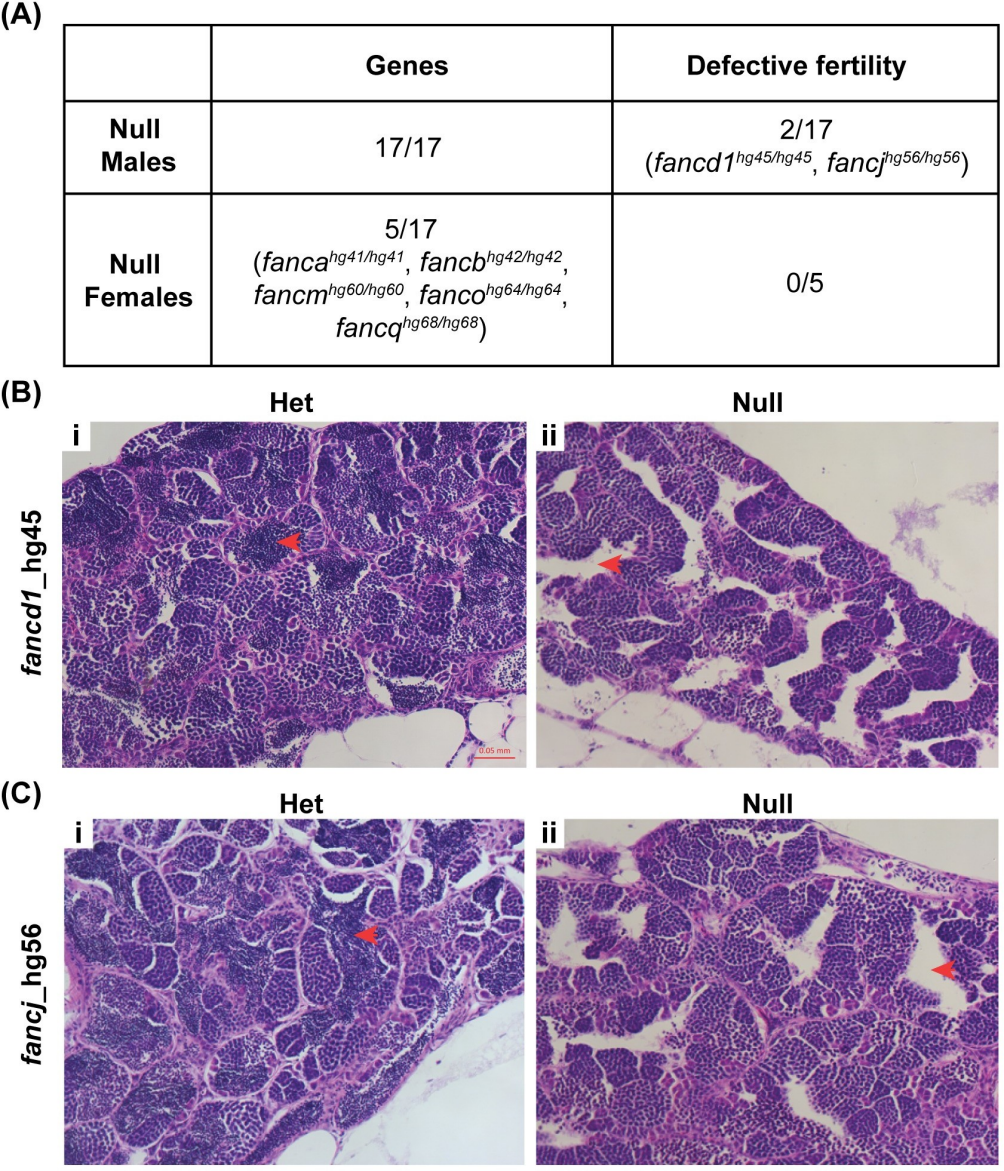
Homozygous knockout fish are fertile except for *fancd1* and *fancj* knockout males. (A) Viability of embryos from outbred homozygous knockout fish at 24 hpf was determined to assess their fertility. Except *fancd1* and *fancj* knockout males, all other knockouts (both males and females) had no fertility defect. For *fancd1*^*hg45/hg45*^ a small fraction of embryos (<5%) were found viable at 24 hpf. (B-C) Histology images (20x objective) of hematoxylin and eosin stained adult testis of *fancd1*^*hg45/hg45*^ (B) and *fancj*^*hg56/hg56*^ fish (C). The red arrows point to the area where mature spermatozoa reside in the testis. Empty intra-testicular ducts were observed in homozygous mutant testis (ii) but not in the heterozygous testis (i).

A critical role played by FA proteins in zebrafish gonadogenesis led us to test whether FA proteins were also required for gametogenesis. Many FA patients experience impaired gametogenesis, defective meiosis and sterility [3, 39]. To this end, we outbred all available FA gene knockout males (all 17 genes) and females (5 genes) with WT fish and checked the embryo viability at 24 hours post fertilization (hpf) to evaluate the fertility of the mutant fish. Surprisingly, the embryos from all but *fancd1*^*hg45/hg45*^ and *fancj*^*hg56/hg56*^ male knockout outbreeding were found viable, indicating knockout male fish for 15 out of 17 FA genes were fertile (Fig 7A). It appears that, but for *fancd1* and *fancj*, all other FA genes may not be necessary for spermatogenesis in zebrafish. Similarly, viable embryos were observed in all five FA gene knockout female outcrosses (*fanca*, *fancb*, *fancm*, *fanco*, *and fancq)* indicating these FA genes are not needed for oogenesis in zebrafish (Fig 7A).

In *fancd1*^*hg45/hg45*^ male outbreeding, only a small fraction of embryos (<5%) were found viable at 24 hpf, indicating a partial sterility phenotype in these fish. With respect to *fancj*^*hg56/hg56*^ male outbreeding, no viable embryos were observed among 25 clutches indicating its essential role in spermatogenesis. To understand the cellular basis for infertility in *fancd1*^*hg45/hg45*^ and *fancj*^*hg56/hg56*^ males, we analyzed their testes by histology. The homozygous knockouts lacked mature spermatozoa in testes, whereas the heterozygous males had normal testes with mature spermatozoa (Fig 7B and 7C).

Presence of complete sterility in *fancj*^*hg56/hg56*^ knockout males prompted us to test the fertility of *fancj*^*hg57/hg57*^ males. Surprisingly, the embryos produced by *fancj*^*hg57/hg57*^ males were viable. The observed contrasting phenotypes between the two *fancj* mutations suggest that one of them may be hypomorphic. The *fancj*^*hg56/hg56*^ fish showed female-to-male sex reversal, the phenotype typical of most FA gene knockouts. The defective fertility phenotype in this line could reflect a critical role of *fancj* in meiotic homologous recombination. This is in contrast to the *fancj*^*hg57/hg57*^ fish that were both fertile and displayed partial sex reversal phenotype, suggesting a potential hypomorphic variant. At the molecular level both alleles are generated from the same sgRNA and are predicted to cause frameshift mutations with premature truncation of the protein, and both alleles display hypersensitivity to DEB treatment (Fig 2A). Thus, our data demonstrate how phenotypes for knockout lines generated from the same CRISPR/Cas9 target site of a gene can vary, emphasizing the importance of testing multiple mutant lines to identify the phenotypic consequences of a gene knockout.

## Discussion

FA is a genetically and phenotypically heterogeneous disorder with mutations in 22 genes known to cause the disease so far. Here, we report generation and characterization of zebrafish knockouts for all recessively inherited FA genes known at the start of this study, except for *FANCS*/*BRCA1* as its zebrafish homolog has not been identified. Three new FA genes (*FANCU*, *FANCV*, *FANCW*) were identified after initiation of our study. A knockout zebrafish model for the only autosomal dominant FA gene (*FANCR/RAD51*) was also reported recently [36]. In addition to the 17 FA genes, our study targeted zebrafish homologs for two genes encoding FA-associated proteins (*FAAP24* and *FAAP100*), which are components of the FA core complex that facilitates the ID2 ubiquitination step of the DNA repair pathway. We generated two loss-of-function alleles for nearly all targeted genes (17/19) enabling evaluation of resulting phenotypes in a reliable manner. In addition, we generated and confirmed viability of eleven combinations of double and triple knockouts, while founder lines to generate many other combinations for multiple gene knockouts are established. This study serves as an integral resource for exploring the FA pathway, and will aid future studies focused on understanding the disease process and the biological processes that become compromised in FA patients, including DNA repair, stem cell maintenance, differentiation of hematopoietic lineages, tumor suppression and aging, among others [1, 6, 14–16, 40].

The ability to perform high throughput CRISPR/Cas9-mediated mutagenesis in zebrafish by a) targeting multiple genes in groups [29, 30], b) utilizing the CRISPR-STAT method to screen for functional guide RNAs [41], and c) adopting a sensitive fluorescent PCR assay for genotyping [42], prompted us to undertake this large effort. We injected seven pools of sgRNA to generate mutant alleles in 19 FA pathway genes. Our data demonstrate that multiplexing of sgRNAs is an efficient approach to generate both individual and multi-gene mutant fish lines. Multiplexing, instead of independent injections, significantly reduced the fish husbandry costs and space needed to establish mutant lines.

To date, zebrafish knockouts for three FA genes, *fancd1*, *fancl* and *fancr/rad51* have been reported, however, none of these employed a targeted mutagenesis approach with one exception. Two independent *fancd1* mutant lines were identified, one caused by retroviral insertion [34] and another by the ENU (N-ethyl-N-nitrosourea)-mediated chemical mutagenesis method [35], both residing in the large exon 11. A recent study reported a *fancd1* mutant line generated by targeting of exon 8 by CRISPR-Cas9 [43]. A *fancl* mutant [33] was identified from an insertion-mutagenesis in a Tol2 transposon-mediated enhancer trap screen. Recently, a knockout for the autosomal dominant FA gene, *fancr/rad51*, was generated using the ENU-mediated method [36]. The emergence of highly efficient CRISPR/Cas9 technology made it possible for us to efficiently test 19 genes.

Our RNA analysis revealed presence of an mRNA carrying the frameshift indel variant in all 36 mutant lines, which is expected to generate truncated proteins. Introducing indel variants in the genomic DNA to create mutant lines can sometime affect splicing by altering the conserved splicing signals such as exonic/intronic splicing enhancers/silencers [44] activating cryptic splice sites. We did observe aberrant splicing in 4/36 mutations, two of which would still result in truncated proteins, while the other two would generate an indel but maintain the reading frame (Fig 1). Often, close proximity of mutations to the natural splice site could affect canonical splicing. Indeed, one of these four (*fancb*_hg42) was located 2bp from the nearest natural splice donor site (S2 Table). Two recent reports also evaluated mRNA expression associated with CRISPR/Cas9-induced indel frameshift mutations in zebrafish and did observe a fraction of aberrantly spliced RNA. Specifically, a 7 bp insertion in *pycr1a* caused exon-skipping leading to 71 bp deletion at the cDNA level [31], and a 7 bp deletion in exon 3 of *smyd1a* resulted in utilization of cryptic splice sites in the adjacent exon [45]. The latter study also identified splicing errors associated with zebrafish missense and nonsense variants resulting in frameshift mutants [45]. Sequence variants in genomic DNA causing aberrant splicing, and thus pathogenesis, are increasingly becoming apparent in genetic diseases [46]. In fact, we have reported instances of aberrant splicing in RNA from FA patients carrying sequence variants in the coding (nonsense, missense, synonymous) and intronic (indel, SNP) [47, 48] regions. Thus, it is important that the consequences of the putative genomic mutations are characterized at the RNA level, which is critical for proper interpretation of the cause and effect of the variants (genotype-to-phenotype).

An ideal way to validate whether a frameshift mutant is indeed null is to demonstrate the absence of the encoded protein. Due to lack of zebrafish antibodies or cross-species reacting antibodies, we could test and show the absence of protein expression in only *fancd2* mutants (S4 Fig). However, adopting a recently reported fluorescent mutation reporter assay [31], we were able to validate several other indel variants, as predicted, did result in premature termination of the reading frame (S5 Fig).

Cellular hypersensitivity to DEB treatment is a hallmark of FA patients. We tested the response of homozygous knockout mutant embryos for all FA genes to DEB treatment by inbreeding heterozygous fish. We could clearly demonstrate the homozygous knockout specific deformed phenotype for seven genes (Fig 2A). Presence of FA genes transcripts as early as one hpf has been reported earlier [27], and the relevance of maternal mRNA and protein in zebrafish embryos has been well documented [49]. This prompted us to speculate that the presence of maternal mRNA may have protected the embryos for some FA gene mutants from displaying the DEB-treatment associated severe malformations. In fact, by incrossing homozygous knockout fish, we did observe the sensitivity of the embryos to DEB treatment. However, this could only be performed for four mutant lines, as female knockout fish were not available for the rest of the FA genes due to female to male sex reversal phenotype (Fig 5). It is interesting to note that malformations were milder for *fancb* mutants (Fig 2C), probably due to a transcript variant that would result in an in-frame protein albeit with insertion of nine amino acids (Fig 1). Altogether, we could test mutant lines for 11/17 FA genes, and demonstrate they are indeed nulls as they all showed DEB hypersensitivity.

Among the FA gene mouse models, embryonic lethality was reported for *Fancd1*, *Fancn*, *and Fanco* knockouts, and for *Fancl* in a specific strain background [50, 51]. This is consistent with FA patients carrying pathogenic mutations in *FANCD1* and *FANCN*, which present with severe phenotype and often die at a young age [52, 53]. All four zebrafish *fancd1* mutant lines [34, 35, 43], including the one in this study, have been found to be viable. A fifth *fancd1* mutant, *zeppelin (zep)*, has been reported recently, which shows lethality between 6 and 10 dpf. The *zep* was isolated in a forward screen for kidney mutants in zebrafish and identified as a homozygous recessive lethal allele that causes reduced podocyte numbers, deficient filtration, and fluid imbalance [54]. The *zep* phenotype was found to be due to a mutation located in a splice acceptor site between exons 20 and 21 resulting in aberrant splicing, and encoding a truncated protein lacking the last 451 amino acids. Unlike the *zep* mutation in intron 20, the mutations in the other four *fancd1* knockouts, including ours, are located in exons 8, 10 or 11. It is tempting to speculate that the larval stage lethality in *zep* may be due to the toxic effects of a truncated protein that lacks the C-terminal DNA binding domain. Alternatively, the non-lethal phenotype in the other three *fancd1* mutants may be due to genetic compensation, hypomorphic alleles, and/or partial complementation by the proteins encoded by the splice variants [55, 56]. We did observe a small fraction of in-frame deletion transcripts in our *fancd1* mutant fish. Only a true *fancd1* null lacking complete expression of RNA would provide an unambiguous reliable null phenotype.

Except for the *fancp*^*-/-*^ fish, where both alleles show smaller body length, visible gross developmental abnormalities were not observed in any other FA gene mutant fish. Co-mutation in *fancq* did not alter this *fancp* phenotype. Incidentally, *Fancp* null mice show similar reduced growth and are born at sub-Mendelian ratios [57]. Growth retardation was observed in mice null for *Fanca*, *Fancc* and *Fancd2* in certain genetic backgrounds [50]. Reduced body size of knockouts of *FANCP* orthologs in both zebrafish and mice models may mimic the short stature observed in ~65% of FA patients [58]. Patients with *FANCP* mutations are rare, however, all seven patients from five FANCP families reported so far do present with growth retardation [59–61]. It is intriguing that loss-of-function in other FA genes does not result in this phenotype in zebrafish. The lack of other gross developmental changes in FA knockout zebrafish models in our study, or by the other groups, could be due to several reasons: redundancy of the pathway, residual function of the mutant proteins, requirement for concomitant loss-of-function mutation in modifier genes, or lack of environmental challenges in a laboratory setting. Using a DNA damaging chemical challenge to induce gene knockout phenotypes in animal models is often necessary and increasingly adopted in various knockout model studies [62]. Most of the FA mouse KO models, do show genomic instability represented by chromosomal breakage only when the knockouts are exposed to DNA crosslinking agents [50, 51]. Requirement of inactivation of modifier genes was illustrated in mouse *Fancd2* mutants that develop severe phenotype when there is a concomitant loss of genes encoding aldehyde catabolism enzymes, ADH and ALDH [63]. Our FA gene knockouts should serve well in future studies in evaluating the effect of modifier genes.

We found that female-to-male sex reversal was a common phenotype in FA gene knockouts (Fig 5), which suggests that FA pathway plays an important role in zebrafish gonadogenesis. Interestingly, complete female-to-male sex reversal was reported in *fancd1*, *fancl* and *fancr* knockout fish, and concomitant knockout of *tp53* rescued the sex reversal phenotype in all three FA gene knockouts [33–36]. In fact, the sex reversal was demonstrated to be due to increased Tp53-mediated germ cell apoptosis at the critical time during sexual fate determination [33]. We also observed rescue of female-to-male sex reversal phenotype when *tp53* null mutation was introduced into *fancp* knockout fish (Fig 6). Hence, it is tempting to speculate that the sex reversal we observed in all FA gene knockouts may also be due to Tp53-mediated apoptosis of germ cells. Since the sex reversal phenotype was present in all of our mutant lines suggests that the mutants are significantly affecting gene function, consistent with the demonstration that these mutants are indeed null alleles.

In FA patients, problems associated with gonadogenesis such as hypogonadism, and infertility are common, particularly male infertility [3, 64], and a recent study identified biallelic loss-of-function *FANCM* mutations as cause of non-obstructive azoospermia [3, 64]. Previous reports in zebrafish have shown that the homozygous knockouts of *fancd1* [34, 35] and *fancr* [36] develop as infertile males with meiotic arrest in spermatocytes. It is surprising that infertility is not a common phenotype in many other FA gene knockout fish. Our *fancd1* mutants confirm the findings from other two mutants but the male infertility phenotype was incomplete. Unlike the complete sterility observed in previously reported *fancd1*^*-/-*^ males, the partial sterility phenotype in our *fancd1*^*hg45/hg45*^ male fish could be due to limited complementation by the less abundant cryptic splice variant predicted to encode a protein lacking 29 amino acids (Fig 1D and 1E). Differences in the level of sterility in *fancd1* knockouts could also result from different target mutation sites (exon 10 in our study *vs* exons 8 and 11 in others). We also report infertility in *fancj*^*-/-*^ males but in only one of the two alleles, and the molecular basis for this allelic discrepancy is not clear.

Our data clearly demonstrate how phenotypes for a gene knockout can vary for different mutant alleles located at a target site. Hence, caution in interpreting any phenotype obtained from a single mutant allele is warranted, underscoring the importance of testing multiple mutant alleles, ideally at different target sites, to identify a legitimate phenotype for a gene knockout. Based on our data and others, it appears that three FA gene knockouts in zebrafish (*fancd1*, *fancj*, and *fancr*) lead to male infertility phenotypes [33, 34, 36]. Interestingly, all three genes participate in the homologous recombination process of the FA pathway, suggesting the recombination repair process mediated by the FANC proteins is active during germ cell development, particularly during meiosis, and defects in this activity can lead to infertility. Our study showed that FA pathway genes play a major role in zebrafish gonadogenesis rather than in gametogenesis, suggesting that hypogonadism among FA patients may lead to the observed increased infertility.

CRISPR/Cas9 mediated genome editing is a double strand break repair process, either by non-homologous end joining (NHEJ) or by homologous recombination. The former is exploited for mutagenesis, while the latter has a higher potential for therapeutic intervention. A very recent report demonstrates that homologous recombination repair by single-strand template requires FA pathway genes [65]. Our mutants can be used for further characterization of this process, and hence can play a role in developing genome-editing based therapeutic approaches.

Taken together, our study adds 32 zebrafish mutant alleles for 17 FA genes using the efficient CRISPR/cas9 technology and extends to encompass nearly all of the known FA genes. These mutant alleles would serve well in the future for exploration of hematopoietic deficiency to understand the bone marrow failure in FA patients. Viability to adult stage observed for all genes enables us to explore biological processes that otherwise would not have been possible as illustrated by the sex reversal and fertility phenotypes. The FA pathway is critical for maintenance of genome integrity, stem cell maintenance and tumor suppression, among others, and we provide resources to study FA pathogenesis as well as to a better understanding of the underlying basic biological functions.

## Materials and methods

### Zebrafish husbandry and Ethics statement

All zebrafish experiments were performed in compliance with the National Institutes of Health guidelines for animal handling and research under NHGRI Animal Care and Use Committee (ACUC) approved protocol G-05-5 assigned to RS and G-17-3 assigned to SCC. Wild-type (WT) zebrafish strain TAB5 was used for all experiments. Zebrafish husbandry, embryo staging and microinjections were performed as described previously [66].

### Generation of knockout mutant zebrafish lines using CRISPR/Cas9

Two single guide RNAs (sgRNAs) per gene, using the criteria indicated in S1 Fig legend, were designed using the ‘ZebrafishGenomics’ track on the UCSC Genome Browser. Synthesis of target oligonucleotides (Integrated DNA Technologies), preparation of mRNA, microinjections, and mutant generation were carried out as described previously [30, 67]. First, CRISPR-STAT was performed to evaluate target-specific activity of one sgRNA per gene as described previously [41]. Next, second sgRNA was tested for the four genes where the first one showed low to no activity. Mutants were generated by microinjections of pooled sgRNAs to multiple genes (1sgRNA/gene chosen based on the CRISPR-STAT data, two to four genes/injection group) based on their known interactions into the yolk of one cell stage embryos [17–19]. The multiplexing scheme along with the targeted exon and sgRNA sequences for each gene are described in Tables 1 and S1. Injected fish were grown to adulthood and screened for germline transmission of indel mutations by breeding with WT fish. High throughput founder screening was performed by analysis of eight embryos per founder fish for indel mutations by fluorescent PCR for each of the genes in the injection group. Sequence of primers used for fluorescent PCR is given in S1 Table. M13F adapter sequence (5’-TGTAAAACGACGGCCAGT) was added to the 5’ end of each forward primer, and PIG-tail sequence (5’-GTGTCTT) was added to the 5’ end of each reverse primer as described [42]. Fluorescent PCR (fPCR) was performed using the gene specific primer pair and a universal FAM-labeled M13F primer (5’-TGTAAAACGACGGCCAGT) as described previously [42, 67]. Same primers were used for CRISPR-STAT, founder screening, identification of heterozygous adult fish from the progeny of selected founders, and for subsequent genotyping to perform genotype-phenotype correlations for all experiments.

### Analysis of expression of mutant alleles by RT-PCR and sequencing

The RT-PCR primers were designed to amplify the exon containing indel mutation along with its flanking exons (S1 Table). The only exception was *fancf*, which is a single exon gene and therefore, as a control we performed no-RT reactions during cDNA synthesis to rule out genomic DNA contamination. Caudal fin tissues from adult WT and homozygous knockout fish for each gene were obtained by ACUC approved fin clip method. RNA was extracted using standard TRI Reagent (Ambion) protocol following tissue homogenization with a Ribolyzer (MP Biomedicals). RNA purification was performed using isopropanol precipitation followed by DNase (Qiagen) treatment. RNA was then passed through Zymo clean and concentrator columns (Zymo Research). Random hexamer primer and 1 μg of total RNA were used to synthesize cDNA using Superscript IV First-Strand Synthesis system for RT-PCR (Invitrogen). Upon completion, the reaction mixture was diluted 1:1 with DEPC water, and 4μl of diluted reaction mixture was used as template for RT- PCR reactions using primers for each of the genes (S1 Table). Amplification of *actb2* (Forward primer– 5’-GTATCCTGACCCTGAAGTACCC-3’; Reverse primer– 5’-AGCACAGCCTGGATGGCAACG-3’) using 2 μl of diluted reaction mixture was performed as control for cDNA quality. The RT-PCR reactions were performed using KAPA2G Fast HotStart ReadyMix PCR Kit (KAPABIOSYSTEMS) as per manufacturer’s instructions, and the products were analyzed on 2% agarose gels. The RT-PCR products were either sequenced directly in case of single band or sequenced after cloning in case of multiple bands. For Sanger sequencing, the PCR products were treated with USB ExoSAP-IT (Affymetrix), and sequencing reactions were carried out with RT-PCR primers using the Bigdye Terminator v3.1 Cycle Sequencing Kit (Applied Biosystems) and run on ABI3730XL sequencer. The sequencing data was evaluated by aligning it to *Danio rerio* reference sequence using Sequencher software (Gene Codes Corporation).

### Functional analysis of frameshift mutant alleles

This assay was performed as described in a recent report [31]. Briefly, primers were designed to amplify the 5’ UTR and a part of coding sequence that includes the mutation site as recommended (S4 Table). RNA was extracted from heterozygous fish and RT-PCR was performed using the SuperScript III One-Step RT-PCR System (Thermo Fisher Scientific) with the following conditions: 50 ^o^C for 30 min, 94 ^o^C for 2 min; 40 cycles of 94 ^o^C for 15 sec, 57 ^o^C for 30 sec, 72 ^o^C for 2 min; 72 ^o^C for 10 min. RT-PCR products were then cloned into the GFP reporter vector and sequence verified to identify WT and mutant clones. As a control, we used the RFP reporter plasmid, pCS2-TagRFPT.zf1 [68]. RNA encoding for GFP and RFP reporters were synthesized using the T3 and SP6 mMessage mMachine kit (Thermo Fisher Scientific) respectively, according to manufacture instructions with LiCl precipitation. For each mutation, we injected a mix of RNA for the WT-GFP or mutant-GFP reporter (200pg) with the control RFP reporter (100pg) into 1 cell embryos. Embryos were imaged at 1dpf on a Leica M205 with a Leica DFC7000GT camera and LAS X Imaging Software Suite.

### Analysis of mutant fish for adult viability and presence of both sexes

The embryos generated from pairwise breeding of single gene and multi-gene heterozygote mutant fish were grown to adulthood (3–6 months). Fin clips from adult fish were processed for DNA extraction using the “Extract-N-Amp” kit (Sigma-Aldrich) and used for genotyping by fluorescent PCR method as described [67]. The genotyping data were used to analyze for Mendelian ratios of surviving homozygous knockout fish compared to the homozygote WT and heterozygous fish. Under the null hypothesis of no viability selection, progeny genotypes should conform to an expected Mendelian ratio of 1:2:1. Deviations from expected number of homozygous knockouts (25%) were tested with goodness-of-fit Chi-square statistical analysis. If the parent fish were heterozygote for mutations in more than one gene, data were analyzed for survival of all possible genotypes expected from these breeding. To get sufficient number of fish genotyped, we analyzed progenies from two breeding for most alleles. To determine the presence of both sexes among surviving adults, all genotyped fish were categorized as males and females and counted.

### Analysis of fertility of mutant fish

The fertility of homozygous knockout fish for each gene was assessed by breeding them with WT fish. The embryo viability was determined at 24 hpf. If the embryos were viable, 7 embryos were collected to confirm their genotype.

### Analysis of body length in *fancp*, and *fancp-fancq* mutant lines

Progenies from inbred single or double heterozygote mutant fish were grown to perform standard body length measurements at indicated time period as described [69]. The juvenile fish at 1 mpf were euthanized to perform standard body length measurements and tissue collection for genotyping as described earlier. Adult fish (4 mpf) were genotyped by fin clipping and measured. Standard body length data was grouped based on their genotype and subjected to one-way ANOVA analysis.

### Histological analyses

Zebrafish were euthanized and fixed in 4% formaldehyde at 4°C for a minimum of 24 hours followed by dehydration in 70% ethanol. Specimens were processed for paraffin embedding and preparation of 5 μm H&E-stained sections (Histoserv). Histological section images were captured with an AxioPlan-2 microscope with AxioCam CCD camera (Zeiss) using ZEN imaging software (Zeiss).

### DEB treatment of embryos

The embryos obtained from indicated breeding crosses were treated with DEB (Sigma Aldrich) at indicated concentrations in egg water with methylene blue between 4 and 72 hpf. The embryos obtained from heterozygous mutant crosses were separated at the end of treatment into three groups based on the severity of the observed morphological changes (normal, moderate and severe) and genotyped using fPCR as described earlier. Representative images of DEB treated embryos obtained from homozygous mutant inbreeding or outbreeding were taken using LAS X Imaging software on a Leica M205 microscope with a DFC7000 color camera.

### Western blot analysis

For Tp53 samples, about twenty-five embryos collected at 24 hpf were dechorionated, deyolked and homogenized in RIPA buffer containing protease inhibitor cocktail (Thermo Fisher Scientific). For Fancd2 samples, soft tissue such as heart, liver, kidney and testis from adult fish were homogenized using TissueRuptor (Qiagen) in cell lysis buffer (Cell Signaling Technology) containing protease inhibitor cocktail (Thermo Fisher Scientific). The homogenates were centrifuged to pellet and remove cellular debris. The samples were resolved on SDS-PAGE (4–15% TGX gels, Bio-Rad) and transferred onto nitrocellulose membrane (Invitrogen). The antibodies for zebrafish Tp53 (Abcam; ab77813), human FANCD2 (Novus Biologicals; NB100-182) and β-actin (Abcam; ab6276) were used at a 1:200, 1:2000 and 1:300 dilutions, respectively.

## Supporting information

S1 Fig Schematic of human and zebrafish FA and FAAP proteins targeted in the study. For each targeted gene, human protein marked with known domains (top) and zebrafish protein marked with the targeted site (red line) and predicted mutant amino acid sequences is shown (bottom). Human protein plots were generated using http://www.cbioportal.org/mutation_mapper.jsp. The target site selection criteria included their location in or upstream of a known domain, present in all known isoforms and in a larger exon for design of accurate genotyping primers.
(PDF)

S2 Fig Nucleotide sequences of the WT and mutant alleles used in this study. For each gene partial WT sequence is shown at the top with sgRNA sequence in bold letters and PAM site underlined. In the mutant alleles, indel is highlighted in yellow, deletions depicted by the dashes and insertions by the lower-case letters. The numbers in parentheses on the right indicate the position in the open reading frame of the sequence shown.
(PDF)

S3 Fig RT-PCR confirms indel mutations and identifies aberrant splice variants caused by mutations. (A) RT-PCR products for all gene mutants along with WT control. The amplified products were resolved on 2% agarose gel. RT-PCR was designed to amplify the exon containing the indel mutation in knockouts and wild-type fish. Minus RT control was performed for hg50 and hg51 lines due to *fancf* being a single exon gene. Expected size products were observed for all mutants, except hg41 (*fanca*), hg42 (*fancb*), hg45 (*fancd1*), and hg58 (*fancl*) mutants, as denoted by the red arrows. Amplicons were sequenced to confirm the mutation, and to determine any aberrant splicing. Multiple products were sequenced after cloning into a vector. (B-E) Representative chromatograms for aberrant splice products of *fancl*_hg58 (B), *fanca*_hg41 (C), *fancb*_hg42 (D), and *fancd1*_hg45 (E). RT-PCR primers for *actb2* were used as transcript control.
(TIFF)

S4 Fig Absence of Fancd2 protein expression in *fancd2* knockouts. Western blot analysis of soft tissue extracts from adult *fancd2* knockout mutants using human FANCD2 antibodies. Extracts obtained from WT fish were used as controls. Expression of β-actin was used as loading control.
(TIFF)

S5 Fig Reporter assay for expression of *fance, fancf, fancg, fancl and fanct* frameshift mutants. Representative images at 1 dpf of embryos co-injected with the specified reporter mRNA and TagRFP are shown as RFP (left panel), GFP (middle panel) and merged (right panel). Merged images show co-expression of the reporter (GFP) and the injection control (RFP) as yellow in the WT allele. However, in the mutant allele only the injection control (RFP) is seen as the GFP is absent due to a premature stop created by the frameshift allele.
(PDF)

S6 Fig Homozygous knockout mutant fish for FA genes survive to adulthood. Progeny from inbred heterozygous fish for each allele were genotyped at 3–6 mpf. Data are shown as stacked bar chart, where each bar represents one mutant allele, as marked on the X-axis by the gene name and hg#. Segments on the bar show % of fish in each of the three expected genotypes: +/+, +/-, and -/- as marked on the Y- axis. Numbers in each segment depict the number of fish for each genotype. The survival data for *fancc_hg*44 and *fance_hg*49 reported here are from double mutant fish, as the mutations for *fancc* and *fance* transmitted together due to their close proximity on the same chromosome.
(TIFF)

S7 Fig Homozygous knockout mutant fish for both *faap100* and *faap24* genes survive to adulthood. Progenies from inbred heterozygous fish for each allele were genotyped at 3–6 mpf. Data are shown as stacked bar chart, where each bar represents one mutant allele, as marked on the X-axis by the gene name and hg#. Segments on the bar show % of fish in each of the three expected genotypes: +/+, +/-, and -/- as marked on the Y- axis. Numbers in each segment depict the number of fish for each genotype. Reduced adult survival for homozygous knockout fish was observed in *faap24*_hg75 line (Chi-square analysis, * p < 0.05).
(TIF)

S8 Fig *fancp^-/-^* fish are significantly smaller in size than their siblings. Standard length measurements of fancp_hg67 fish at 1 mpf (A), and 4 mpf (B). (i) Representative images of *fancp*^*+/+*^ and *fancp*^*hg67/hg67*^ fish with red arrows marking the beginning of caudal fin used in length measurements. (ii) Data on body size measurements for *fancp*^*+/+*^, *fancp*^*hg67/+*^ and *fancp*^*hg67/hg67*^ fish. Both time points show a significant decrease in size of *fancp*^*hg67/hg67*^ fish compared to the WT and heterozygous clutch mates (Chi-square analysis, p<0.001).
(TIFF)

S9 Fig Co-mutation in *fancq* gene does not affect the reduced body length of *fancp* homozygous knockouts. Progenies from incrossed *fancp*^*hg66/+*^;*fancq*^*hg68/+*^ fish were used for evaluation at 1 mpf. Standard body length measurements of each juvenile fish are plotted on Y-axis for all possible nine genotypic combinations shown on X-axis. *fancp*^*hg66/hg66*^ homozygous knockouts in combination with all three possible *fancq* genotypes showed significantly decreased body length compared to double WT clutch mates (ANOVA analysis, ***p<0.001, **p<0.01).
(TIFF)

S10 Fig Evaluation of partial sex reversal phenotype in the second mutant allele. Progenies from inbred heterozygous fish for the second mutant allele for each gene were genotyped around 3 mpf and the sex was determined. For each gene, the data is shown as bar graph for number of male and female fish in each genotype category as marked on the X-axis. Numbers at the top of bar depict the number of fish.
(TIFF)

S11 Fig Gonadal development during larval and juvenile stages of mutant fish. Histological sections of gonads from *fancc*_hg43 heterozygotes and homozygotes at 21 dpf (A) and 45 dpf (B). (A) The bipotential gonads of *fancc*^*hg43/+*^ and *fancc*^*hg43/hg43*^ are indistinguishable at 21 dpf. (B) At 45 dpf, the gonads of *fancc*^*hg43/+*^ exhibit continued maturation of testes or ovaries, whereas *fancc*^*hg43/hg43*^ exhibit only testicular development. go, gonocyte; sc, spermatocyte; oc, oocyte.
(TIFF)

S1 Table Multiplexing scheme, targeted exons, sgRNA sequences, genotyping primers, and RT-PCR primers. (XLSX)

S2 Table Description of single gene mutant alleles used in this study. (XLSX)

S3 Table Survival of multiple gene knockout fish to adulthood. (XLSX)

S4 Table Primers for functional analysis of frameshift alleles. (XLSX)
