# Supplementary figures and images for: Multiplexed CRISPR/Cas9-mediated knockout of 19 Fanconi anemia pathway genes in zebrafish revealed their roles in growth, sexual development and fertility
Source: PLoS Genet. 2018 Dec 12;14(12):e1007821. doi: 10.1371/journal.pgen.1007821 (PMC6328202; doi:10.1371/journal.pgen.1007821)

S1 Fig

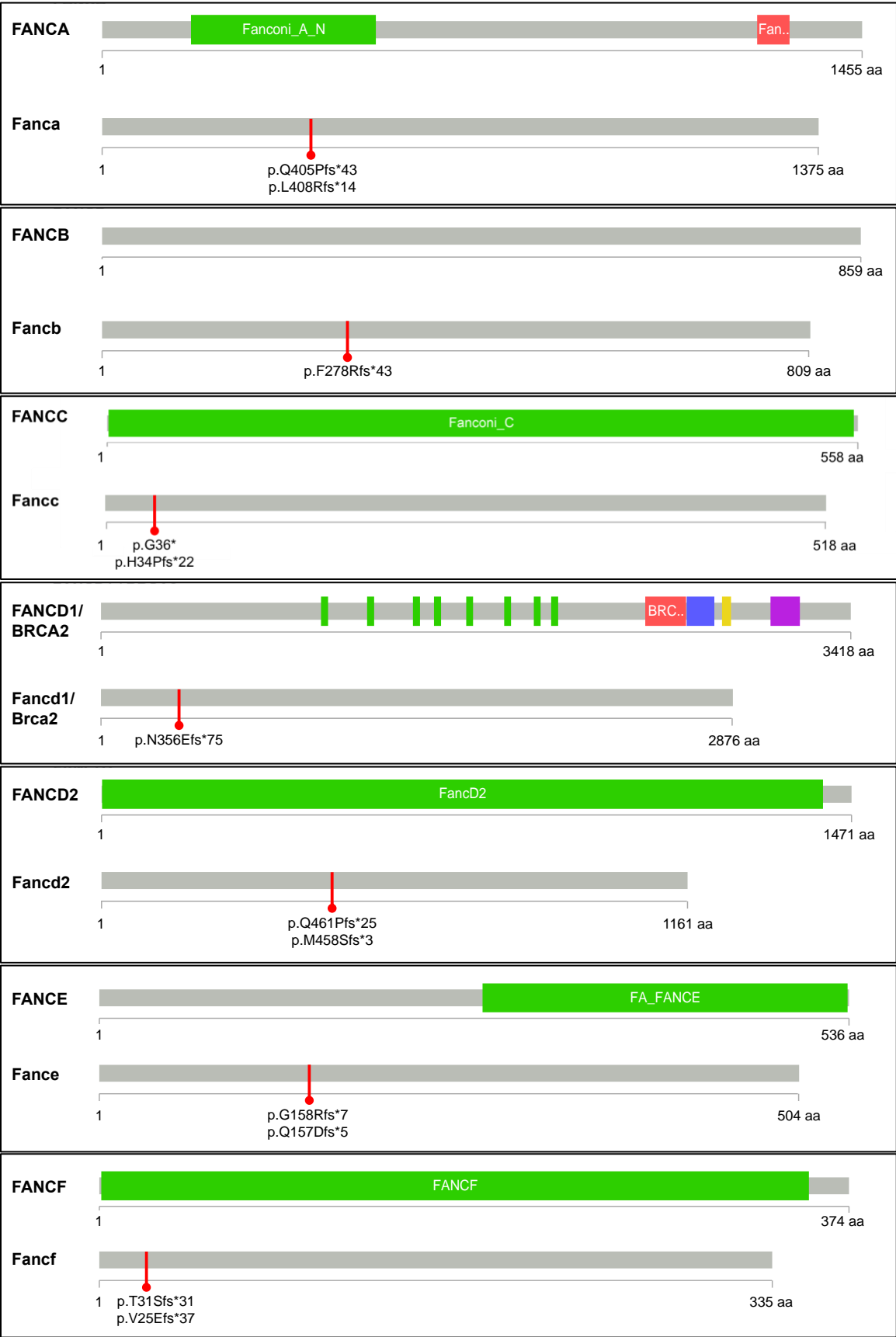

S1 Fig continued

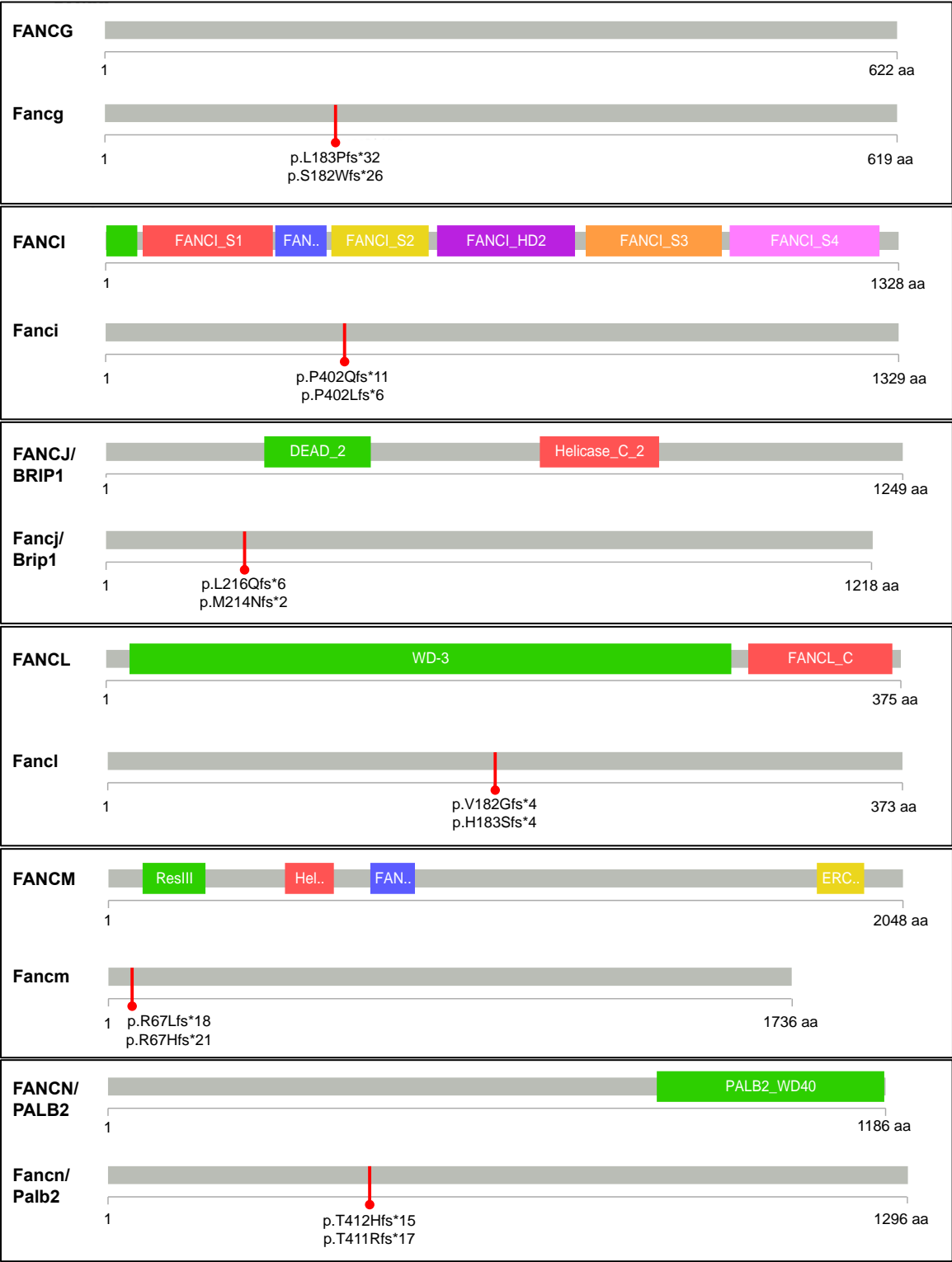

S1 Fig continued

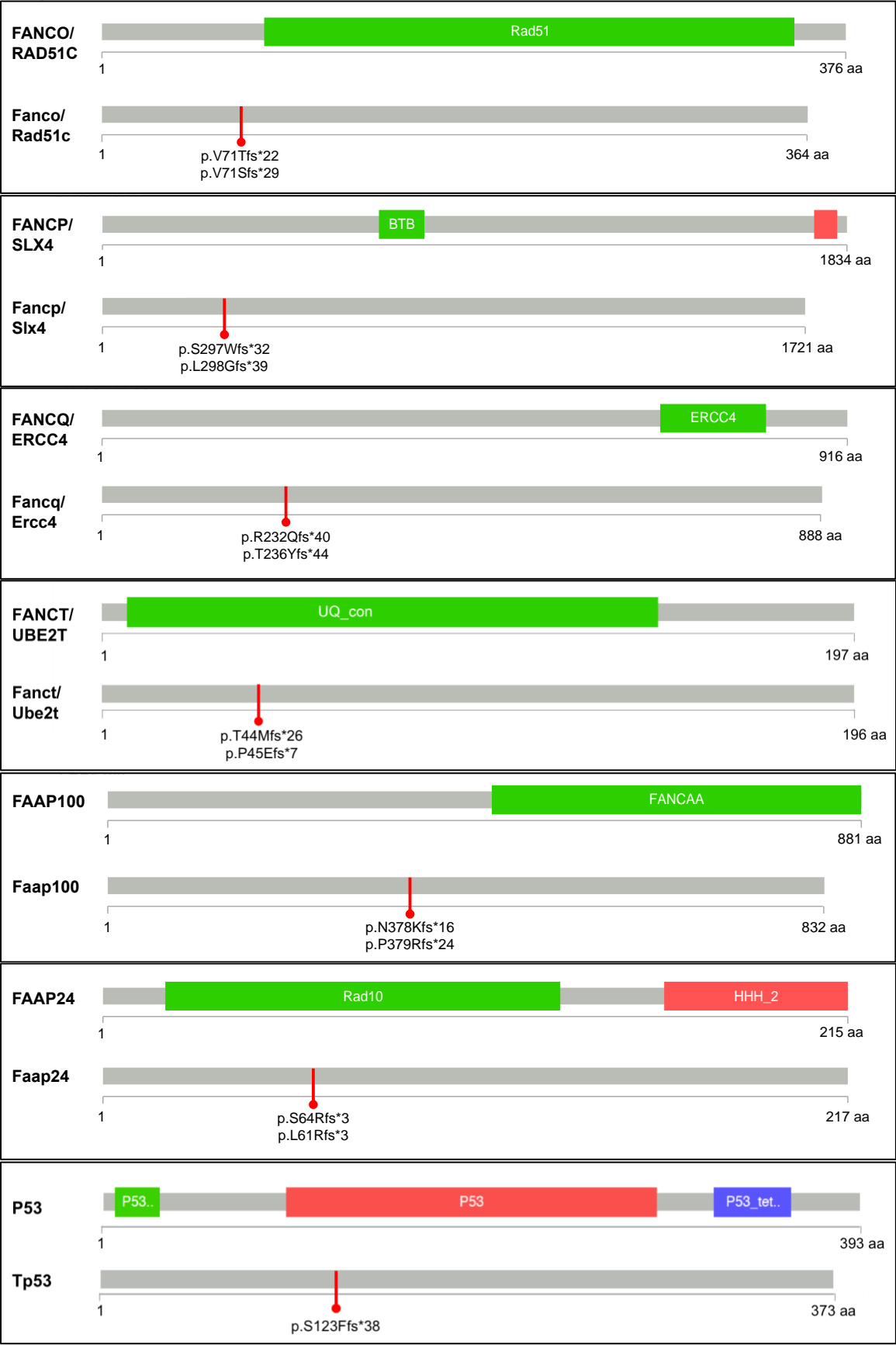

Supplement: S1 Fig — For each targeted gene, human protein marked with known domains (top) and zebrafish protein marked with the targeted site (red line) and predicted mutant amino acid sequences is shown (bottom). Human protein plots were generated using http://www.cbioportal.org/mutation_mapper.jsp. The target site selection criteria included their location in or upstream of a known domain, present in all known isoforms and in a larger exon for design of accurate genotyping primers. (PDF) [file pgen.1007821.s001.pdf]

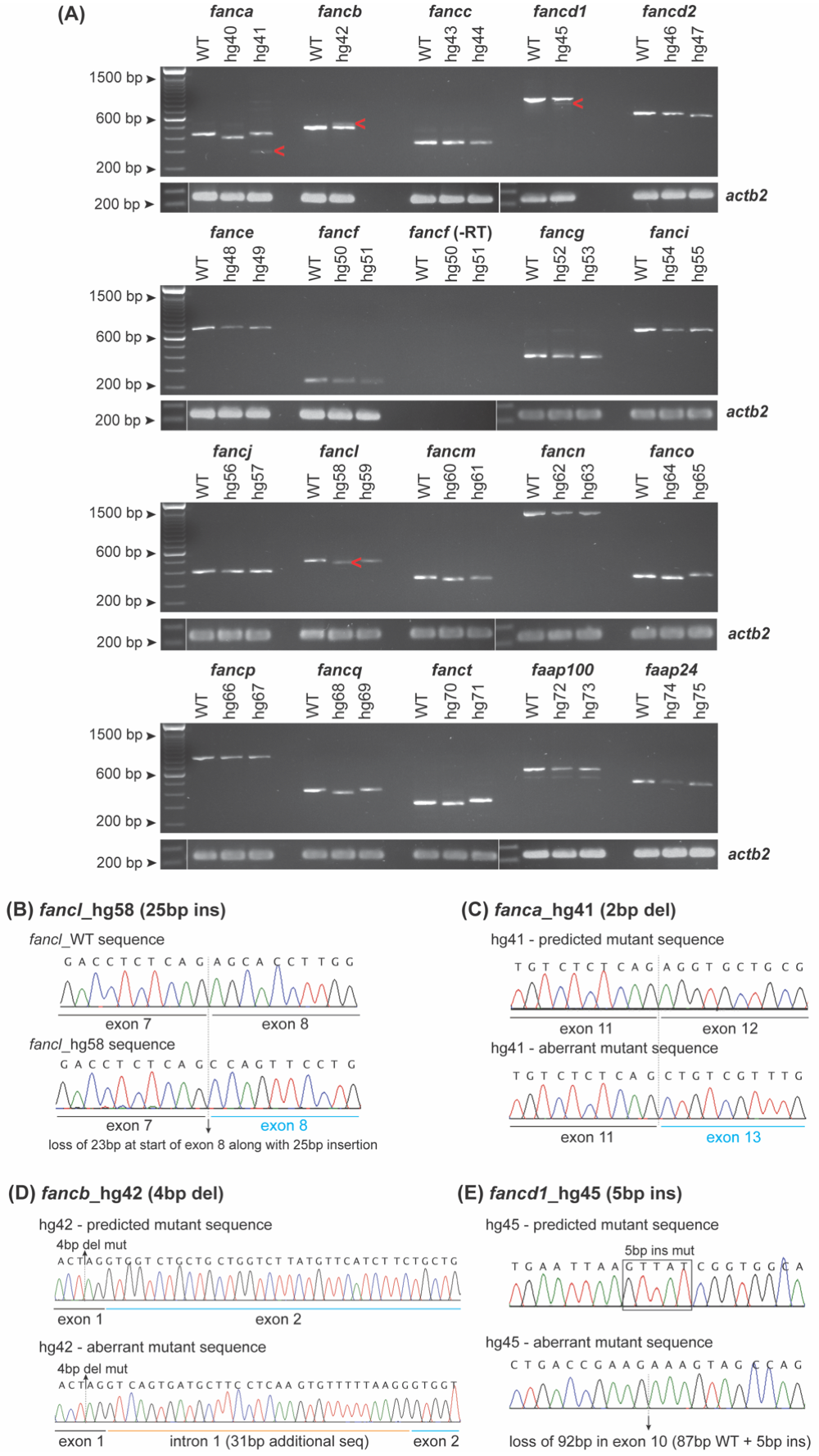

Supplement: S3 Fig — (A) RT-PCR products for all gene mutants along with WT control. The amplified products were resolved on 2% agarose gel. RT-PCR was designed to amplify the exon containing the indel mutation in knockouts and wild-type fish. Minus RT control was performed for hg50 and hg51 lines due to fancf being a single exon gene. Expected size products were observed for all mutants, except hg41 (fanca), hg42 (fancb), hg45 (fancd1), and hg58 (fancl) mutants, as denoted by the red arrows. Amplicons were sequenced to confirm the mutation, and to determine any aberrant splicing. Multiple products were sequenced after cloning into a vector. (B-E) Representative chromatograms for aberrant splice products of fancl_hg58 (B), fanca_hg41 (C), fancb_hg42 (D), and fancd1_hg45 (E). RT-PCR primers for actb2 were used as transcript control. (TIFF) [file pgen.1007821.s003.tiff]

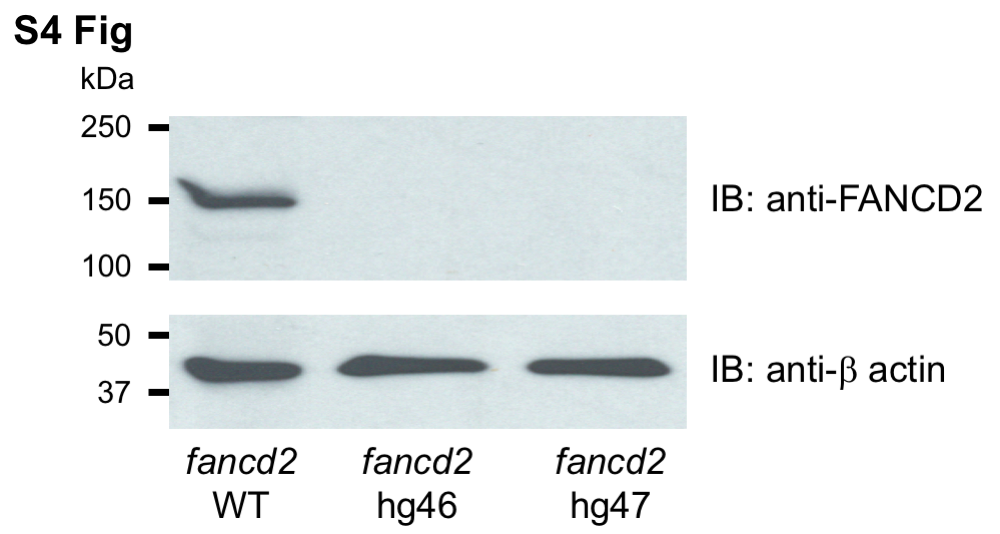

Supplement: S4 Fig — Western blot analysis of soft tissue extracts from adult fancd2 knockout mutants using human FANCD2 antibodies. Extracts obtained from WT fish were used as controls. Expression of β-actin was used as loading control. (TIFF) [file pgen.1007821.s004.tiff]

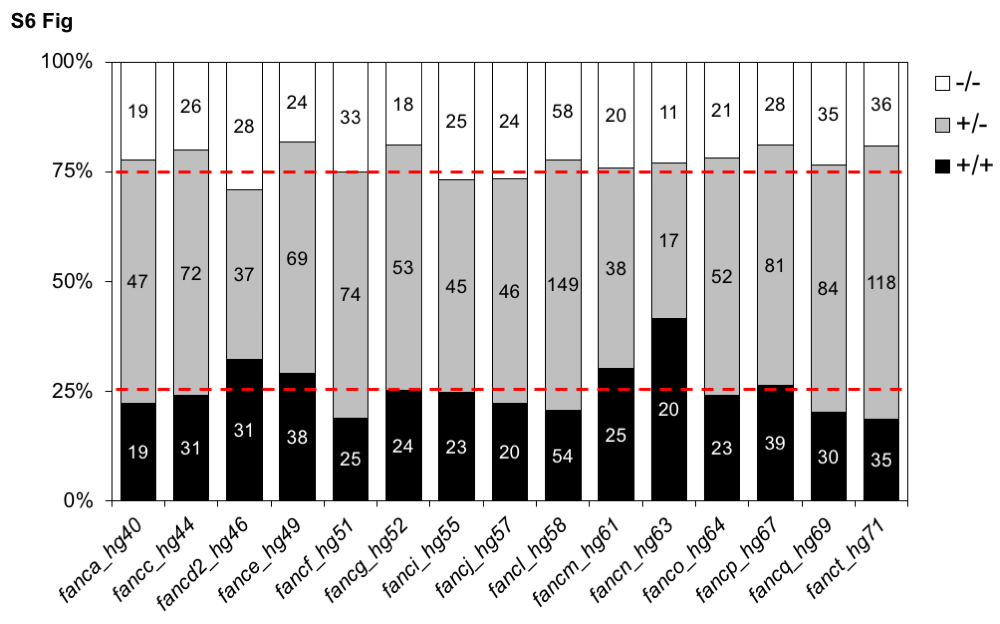

Supplement: S6 Fig — Progeny from inbred heterozygous fish for each allele were genotyped at 3–6 mpf. Data are shown as stacked bar chart, where each bar represents one mutant allele, as marked on the X-axis by the gene name and hg#. Segments on the bar show % of fish in each of the three expected genotypes: +/+, +/-, and -/- as marked on the Y- axis. Numbers in each segment depict the number of fish for each genotype. The survival data for fancc_hg44 and fance_hg49 reported here are from double mutant fish, as the mutations for fancc and fance transmitted together due to their close proximity on the same chromosome. (TIFF) [file pgen.1007821.s006.tiff]

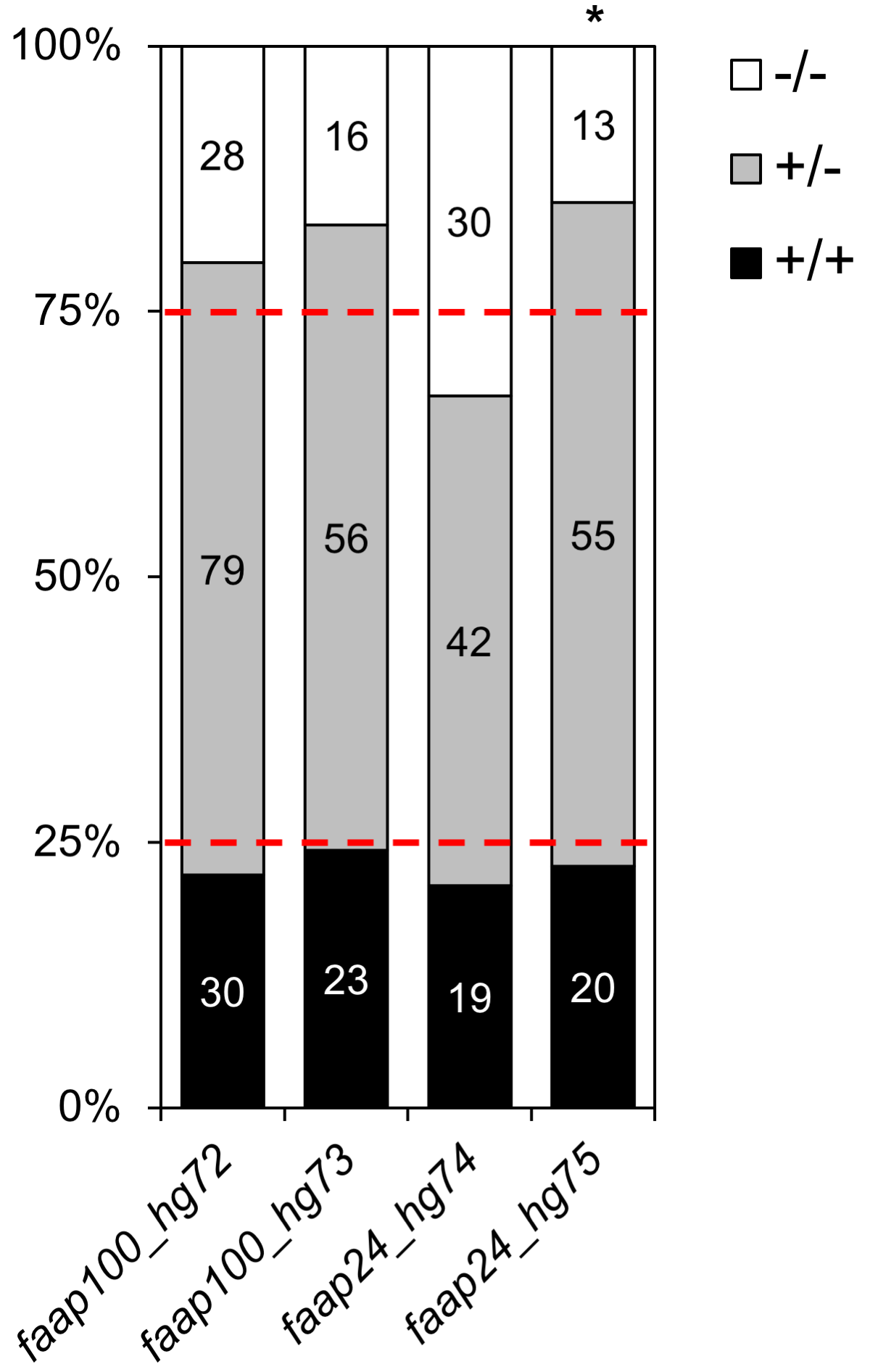

Supplement: S7 Fig — Progenies from inbred heterozygous fish for each allele were genotyped at 3–6 mpf. Data are shown as stacked bar chart, where each bar represents one mutant allele, as marked on the X-axis by the gene name and hg#. Segments on the bar show % of fish in each of the three expected genotypes: +/+, +/-, and -/- as marked on the Y- axis. Numbers in each segment depict the number of fish for each genotype. Reduced adult survival for homozygous knockout fish was observed in faap24_hg75 line (Chi-square analysis, * p < 0.05). (TIF) [file pgen.1007821.s007.tif]

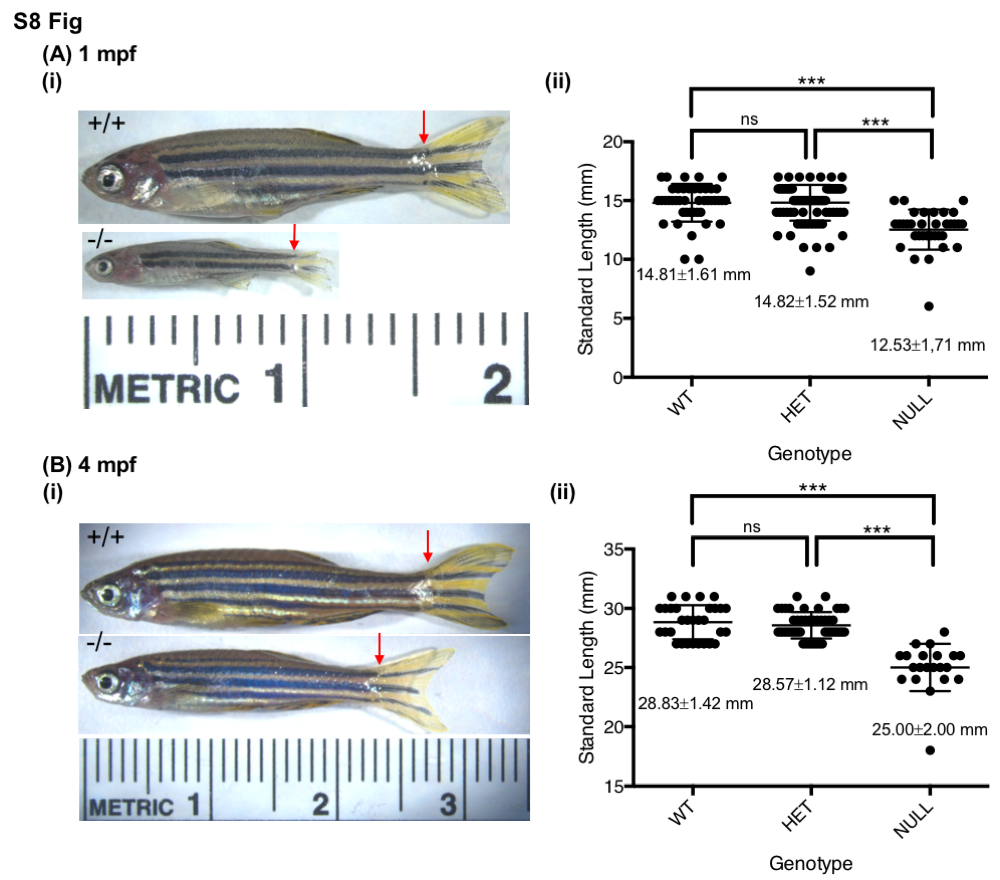

Supplement: S8 Fig — Standard length measurements of fancp_hg67 fish at 1 mpf (A), and 4 mpf (B). (i) Representative images of fancp+/+ and fancphg67/hg67 fish with red arrows marking the beginning of caudal fin used in length measurements. (ii) Data on body size measurements for fancp+/+, fancphg67/+ and fancphg67/hg67 fish. Both time points show a significant decrease in size of fancphg67/hg67 fish compared to the WT and heterozygous clutch mates (Chi-square analysis, p<0.001). (TIFF) [file pgen.1007821.s008.tiff]

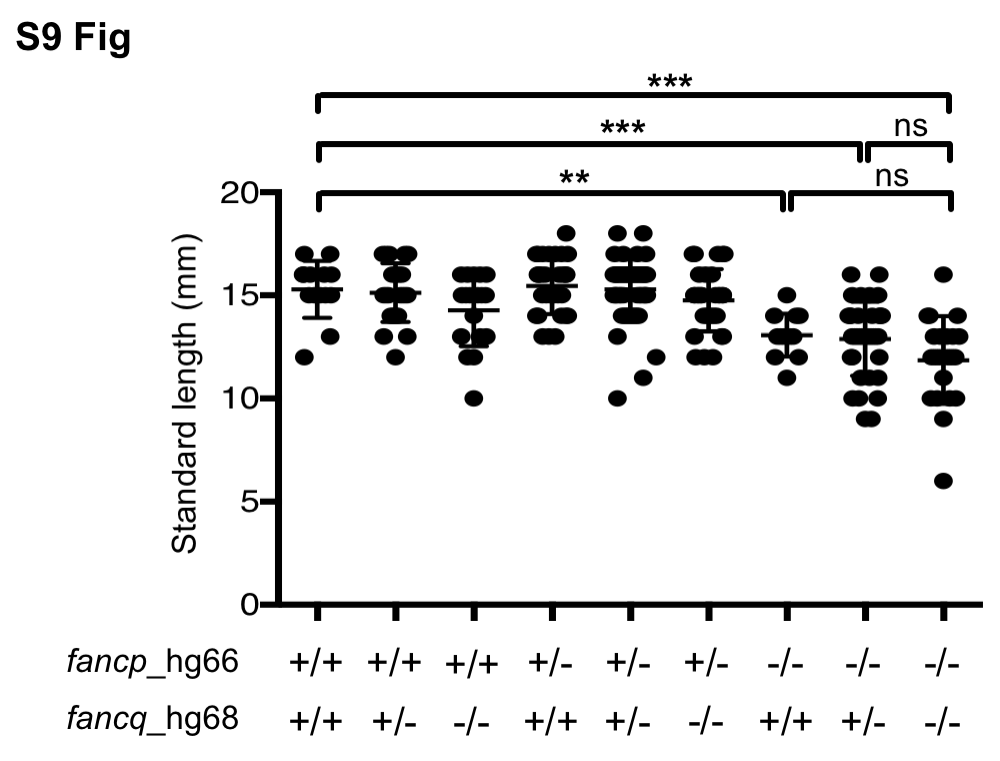

Supplement: S9 Fig — Progenies from incrossed fancphg66/+;fancqhg68/+ fish were used for evaluation at 1 mpf. Standard body length measurements of each juvenile fish are plotted on Y-axis for all possible nine genotypic combinations shown on X-axis. fancphg66/hg66 homozygous knockouts in combination with all three possible fancq genotypes showed significantly decreased body length compared to double WT clutch mates (ANOVA analysis, ***p<0.001, **p<0.01). (TIFF) [file pgen.1007821.s009.tiff]

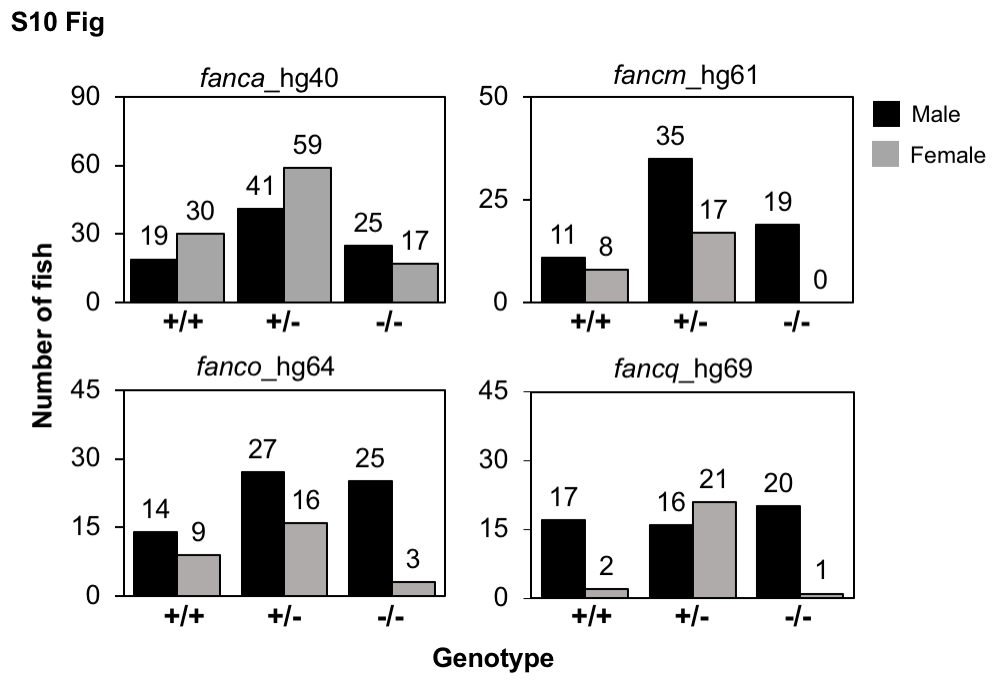

Supplement: S10 Fig — Progenies from inbred heterozygous fish for the second mutant allele for each gene were genotyped around 3 mpf and the sex was determined. For each gene, the data is shown as bar graph for number of male and female fish in each genotype category as marked on the X-axis. Numbers at the top of bar depict the number of fish. (TIFF) [file pgen.1007821.s010.tiff]

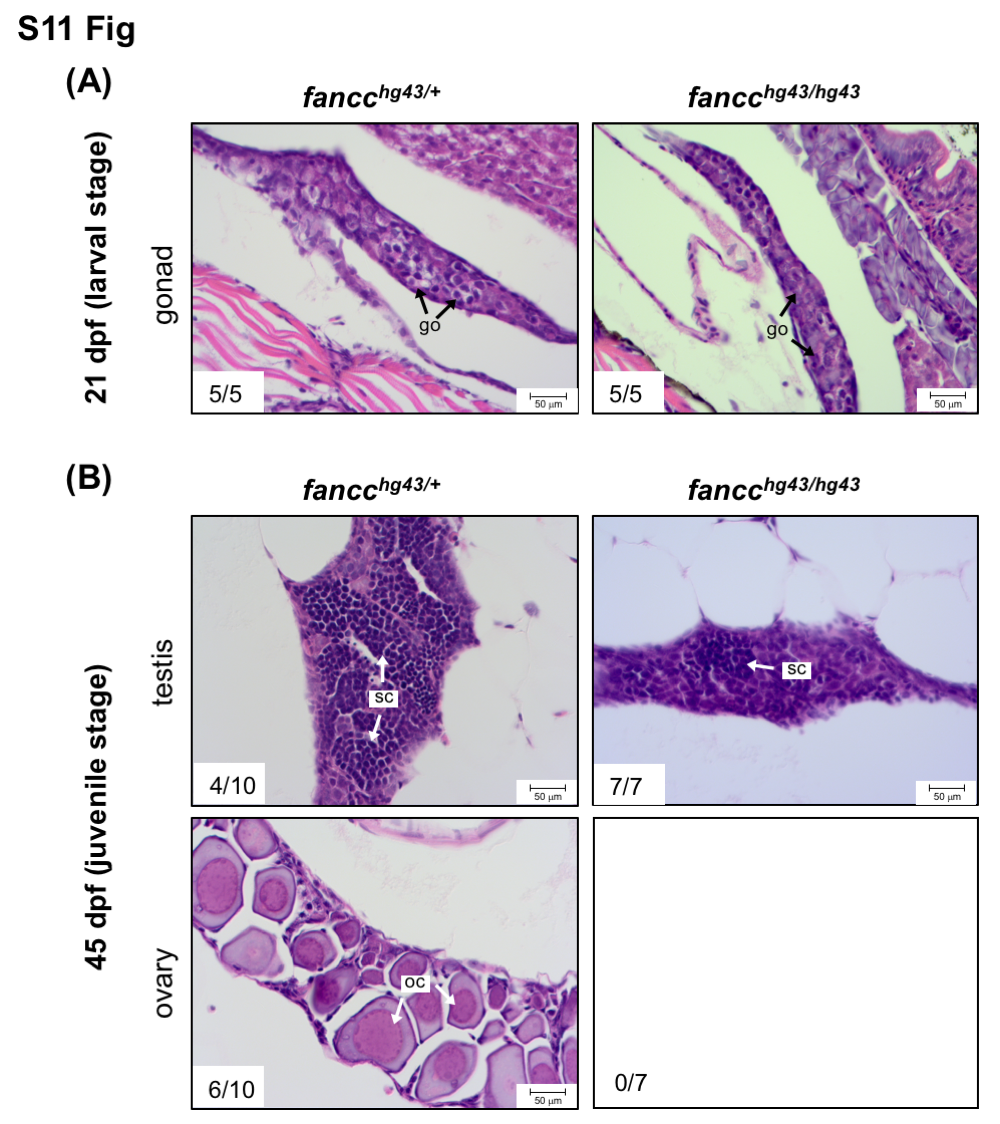

Supplement: S11 Fig — Histological sections of gonads from fancc_hg43 heterozygotes and homozygotes at 21 dpf (A) and 45 dpf (B). (A) The bipotential gonads of fancchg43/+ and fancchg43/hg43 are indistinguishable at 21 dpf. (B) At 45 dpf, the gonads of fancchg43/+ exhibit continued maturation of testes or ovaries, whereas fancchg43/hg43 exhibit only testicular development. go, gonocyte; sc, spermatocyte; oc, oocyte. (TIFF) [file pgen.1007821.s011.tiff]
