# Supplementary material for: Multiplexed CRISPR/Cas9-mediated knockout of 19 Fanconi anemia pathway genes in zebrafish revealed their roles in growth, sexual development and fertility
Source: PLoS Genet. 2018 Dec 12;14(12):e1007821. doi: 10.1371/journal.pgen.1007821 (PMC6328202; doi:10.1371/journal.pgen.1007821)
Supplement: S2 Fig — For each gene partial WT sequence is shown at the top with sgRNA sequence in bold letters and PAM site underlined. In the mutant alleles, indel is highlighted in yellow, deletions depicted by the dashes and insertions by the lower-case letters. The numbers in parentheses on the right indicate the position in the open reading frame of the sequence shown. (PDF) [file pgen.1007821.s002.pdf]

S2 Fig

fanca		
WT	T**GGCGCGGCAGGCGGCTCTCG**AGGGCCCAGCG//TCCAGTCC	(1205-1248)
Δ34 (hg40)	TGGCGCG-------------------------//-----TCC	
Δ2 (hg41)	TGGCGCGGCAGGCGGCTC--GAGGGCCCAGCG	
fancb		
WT	ATGTCTGCGC**GGTGTGGAAGGACACTTTCC**AGGTGGTCTG	(806-845)
Δ4 (hg42)	ATGTCTGCGCGGTGTGGAAGGACACT----AGGTGGTCTG	
fancc		
WT	CAGCTCTTCT**GGACACTAGCAGACACCT**TGGCCCTCTGAG	(77-116)
Δ1 (hg43)	CAGCTCTTCTGGACACTAGCAGACAC-TTGGCCCTCTGAG	
Δ2 (hg44)	CAGCTCTTCTGGACACTAGCAGAC--CTTGGCCCTCTGAG	
fancd1		
WT	ACTTCTTT**GGTAGACAATAACAACACGG**TGGCACCCA	(1048-1084)
+5bp (Δ7, +12; hg45)	ACTTCTTTGGTAGACAATgaattaagttatCGGTGGCACCCA	
fancd2		
WT	GCCTTTC**GGGGGTCACATGTACAAAC**AGGCCTTCACTGCCT	(1356-1396)
Δ4 (hg46)	GCCTTTCGGGGGTCACATGTACA----GGCCTTCACTGCCT	
Δ20 (hg47)	GCCTTTCGGGGGTCACA--------------------GCCT	
fance		
WT	GGAAGATTTC**GGGGTACTGATGTACAGGGA**GGGTGGACAT	(445-484)
Δ2 (hg48)	GGAAGATTTCGGGGTACTGATGTACAG--AGGGTGGACAT	
Δ11 (hg49)	GGAAGATTTCGGGGTACTGA-----------GGTGGACAT	
fancf		
WT	GTGCGCGAAT**GGGACCGTCCCACCACACAG**CGGGCGTTTA	(67-106)
Δ4 (hg50)	GTGCGCGAATGGGACCGTCCCACCA----GCGGGCGTTTA	
Δ10 (Δ23, +13; hg51)	GTGCGCG---taagaaaatggga-------CGGGCGTTTA	
fancg		
WT	CTCTGAGACT**GGAAGAAGACACCTCACTAA**TGGTTGCCAT	(521-560)
Δ5 (Δ7, +2; hg52)	CTCTGAGACTGGAAGAAGACACCTCAC--ca---TGCCAT	
Δ7 (hg53)	CTCTGAGACTGGAAGAAGACAC-------ATGGTTGCCAT	
fanci		
WT	CTCATTC**GGCCCTAAACCAGGACCATT**CGGAAAGGTG	(1182-1218)
Δ8 (hg54)	CTCATTCGGCCCTAAACCAGGAC--------AAGGTG	
+2 (Δ3, +5; hg55)	CTCATTCGGCCCTAAACCAGGcctaaATTCGGAAAGGTG	
fancj		
WT	ACTTGGACCA**TGGAATTGAACAACGGAGCC**AGAGCTGACA	(631-670)
Δ5 (hg56)	ACTTGGACCATG-----GAACAACGGAGCCAGAGCTGACA	
Δ4 (hg57)	ACTTGGACC----AATTGAACAACGGAGCCAGAGCTGACA	

S2 Fig continued

fancl		
WT	GCCA**ACGTCCACAGCCAGTTCC**T	(538-560)
+25 (hg58)	GCCAACGgattgatatgatttatgttgatggcTCCACAGCCAGTTCCT	
Δ7 (hg59)	GCCAAC-------GCCAGTTCCT	
fancm		
WT	TCCGCCG**GCCGTGTCTGGATCTACCCC**ACCAACCTGC	(190-226)
Δ5 (hg60)	TCCGCCGGCC-----TGGATCTACCCCACCAACCTGC	
+4 (hg61)	TCCGCCGGCCatctGTGTCTGGATCTACCCCACCAACCTGC	
fancn		
WT	GAGGAGA**GGACGTCGAAGAACGACTGT**AGGACCCACA	(1212-1248)
Δ11 (hg62)	GAGGAGAGGACGTCGAAGAAC-----------CCACA	
Δ8 (hg63)	GAGGAGAGGACGTCGAAGAA--------GGACCCACA	
fanco		
WT	AGAG**GGCAGCAGCAGACGGCGTAA**CGGCACT	(191-221)
Δ8 (hg64)	AGAGGGCAGCAGCAG--------ACGGCACT	
+13 (Δ1, +14; hg65)	AGAGGGCAGCAGCAGACGGCagtggatcaatataTAACGGCACT	
fancp		
WT	GGCGCT**GGCTCTGTCCCGCTCTCTGC**TGGAGCAGGA	(870-905)
Δ7 (hg66)	GGCGCTGGCTCTGTCCCGCT-------GGAGCAGGA	
Δ5 (hg67)	GGCGCTGGCTCTGTCCCGCTCT-----GGAGCAGGA	
fancq		
WT	CGCTACAACCC**CACACTGGAAGCAGAAGACC**TTTCT	(694-729)
Δ22 (hg68)	C----------------------AGAAGACCTTTCT	
+2 (Δ2, +4; hg69)	CGCTACAACCCCACtacaTGGAAGCAGAAGACCTTTCT	
fanct (ube2t)		
WT	TGGA**GGTGCAAACACTCCGTATGA**AGGTGGTG	(217-248)
Δ7 (hg70)	TGGAGGTGCAAACA-------TGAAGGTGGTG	
+13 (Δ4, +17; hg71)	TGGAGGTGCAAACACTgaaggtgaagtgtctggATGAAGGTGGTG	
faap100		
WT	ACTTGCT**GGACCATATACAAACCCAACA**GGGTCCGTT	(1113-1149)
Δ4 (hg72)	ACTTGCTGGACCATATACAA----AACAGGGTCCGTT	
Δ5 (hg73)	ACTTGCTGGACCATATACA-----AACAGGGTCCGTT	
faap24		
WT	GTGTCCT**CTATGTGTCAGAAAGTGACC**TGGTGGCAGG	(176-212)
Δ2 (hg74)	GTGTCCTCTATGT--CAGAAAGTGACCTGGTGGCAGG	
Δ11 (hg75)	GTGTC-----------AGAAAGTGACCTGGTGGCAGG	
tp53		
WT	CCTCCACAGGGCTCCG**TGGTTCGAGCCACTGCCATC**TA	(355-392)
Δ7 (hg91)	CCTCCACAGGGCT-------TCGAGCCACTGCCATCTA	
