# Supplementary material for: Multiplexed CRISPR/Cas9-mediated knockout of 19 Fanconi anemia pathway genes in zebrafish revealed their roles in growth, sexual development and fertility
Source: PLoS Genet. 2018 Dec 12;14(12):e1007821. doi: 10.1371/journal.pgen.1007821 (PMC6328202; doi:10.1371/journal.pgen.1007821)

S5 Fig

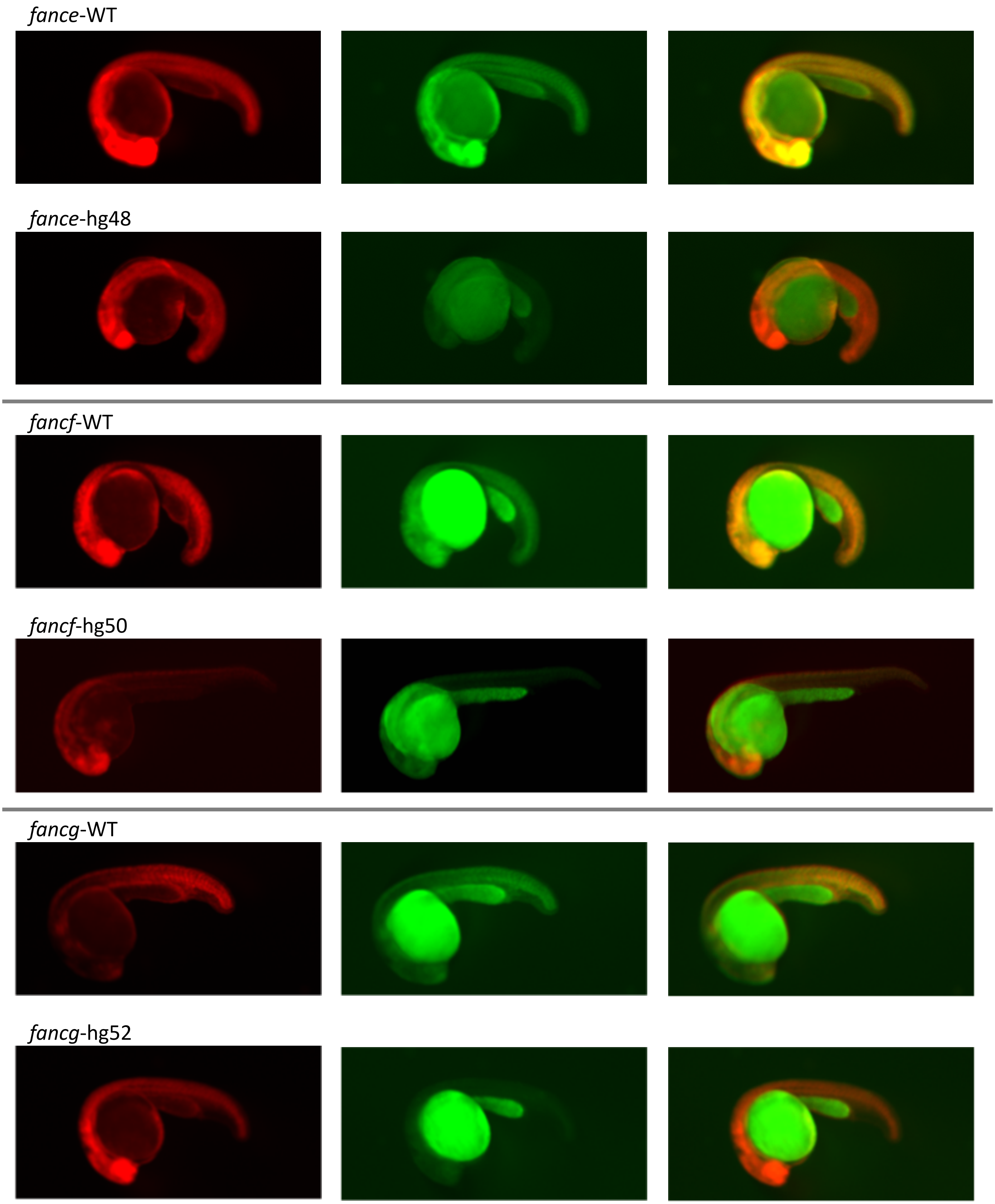

S5 Fig continued

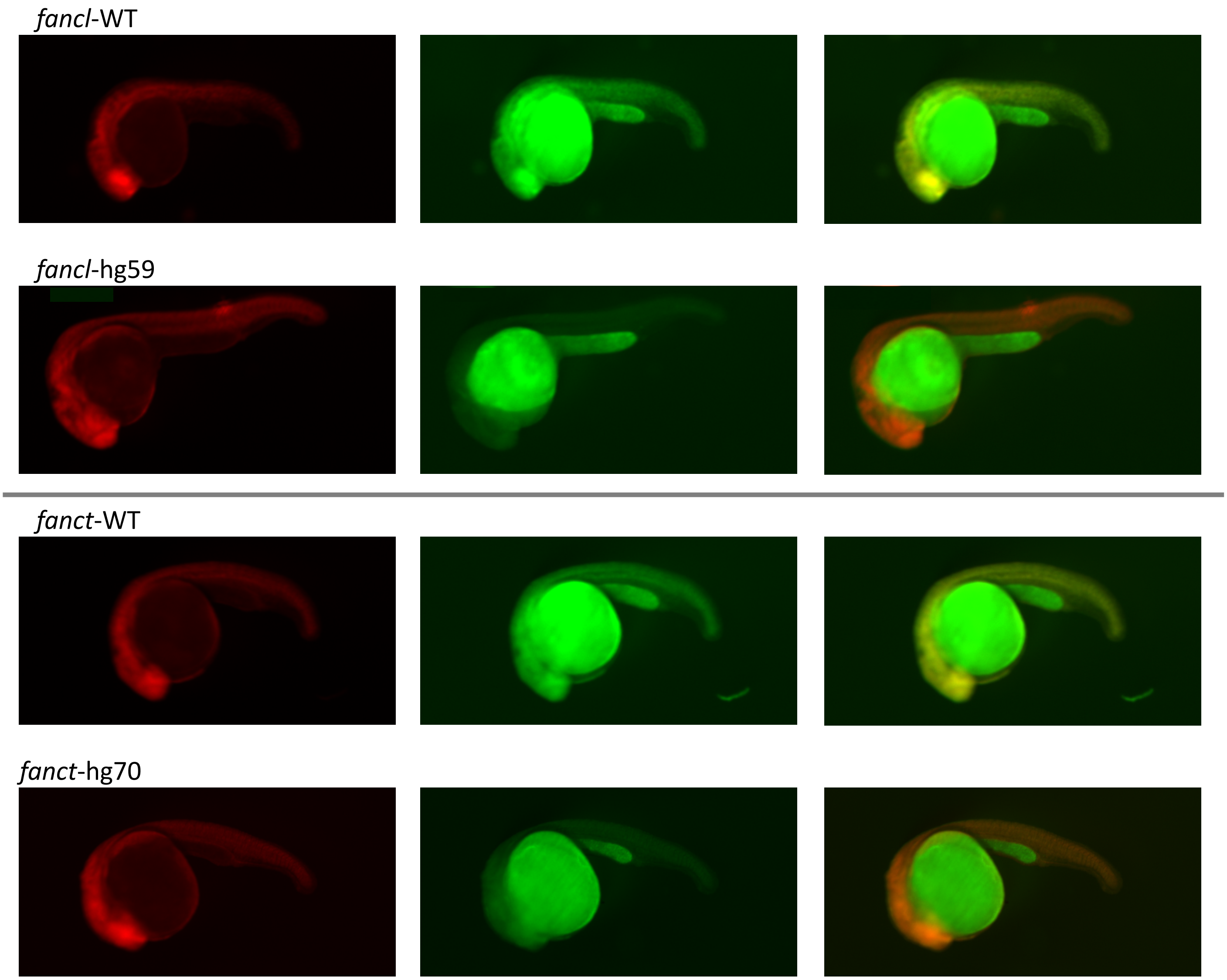

Supplement: S5 Fig — Representative images at 1 dpf of embryos co-injected with the specified reporter mRNA and TagRFP are shown as RFP (left panel), GFP (middle panel) and merged (right panel). Merged images show co-expression of the reporter (GFP) and the injection control (RFP) as yellow in the WT allele. However, in the mutant allele only the injection control (RFP) is seen as the GFP is absent due to a premature stop created by the frameshift allele. (PDF) [file pgen.1007821.s005.pdf]
